# Supplementary material for: Estimating the Impact of Consecutive Blood Meals on Vector Competence of Aedes albopictus for Chikungunya Virus
Source: Pathogens. 2023 Jun 20;12(6):849. doi: 10.3390/pathogens12060849 (PMC10303208; doi:10.3390/pathogens12060849)
Supplement: Supplementary file 1 [file pathogens-12-00849-s001.zip › pathogens-2408036-supplementary 1.pdf]

Table S1. Description of feeding rate and survival rate for *Ae. albopictus* females from the control (only one infectious meal) and second group (exposed to a second non-infectious blood meal).

Exposed to blood	Engorged*	Temperature regime	Survived (4 dpi)	Engorged	Survived (7 dpi)	Survived (10 dpi)
**Control**						
66	39 (59%)	CT	n/a	n/a	30 (77%)	-
87	44 (51%)	CT	n/a	n/a	-	25 (57%)
123	82 (67%)	FT	n/a	n/a	29 (35%)	-
62	40 (65%)	FT	n/a	n/a	-	31 (78%)
**2nd blood meal**						
185	105 (57%)	CT	97	32 (33%)	20 (63%)	-
308	221 (71%)	CT	203	66 (33%)	-	30 (44%)
255	167 (65%)	FT	140	56 (40%)	30 (54%)	-
250	138 (55%)	FT	128	38 (30%)	-	30 (79%)

*these totals do not include day 0 females
