# Supplementary material for: Estimating the Impact of Consecutive Blood Meals on Vector Competence of Aedes albopictus for Chikungunya Virus
Source: Pathogens. 2023 Jun 20;12(6):849. doi: 10.3390/pathogens12060849 (PMC10303208; doi:10.3390/pathogens12060849)
Supplement: Supplementary file 1 [file pathogens-12-00849-s001.zip › pathogens-2408036-supplementary 2.pdf]

# Graphical Analysis and Modelling: Vector competence Project

Author: Dr. Matteo Tanadini | Zurich Data Scientists
Reviewer: Nisia Trisconi | Zurich Data Scientists

May 31, 2023

## Contents

**1 Freezing Package versions** **3**

**2 Load packages** **3**

**3 Getting data** **3**

**4 Estimating confidence intervals for binomial proportions** **4**

**5 Graphical Analysis** **7**
5.1 Proto-plot . . . 7
5.2 Infection rate . . . 7
5.3 Dissemination efficiency . . . 8
5.4 Dissemination rate . . . 9
5.5 Transmission efficiency . . . 10
5.6 Transmission rate . . . 11
5.7 PFU . . . 12
5.8 All graphs together . . . 14

**6 PFU (formal modelling)** **16**
6.1 PFU (visualising model fit) . . . 19

**7 Methods description** **20**

**8 Figures for scientific publication** **20**
8.1 Infection rate . . . 20
8.2 Dissemination efficiency . . . 21
8.3 Dissemination rate . . . 22
8.4 Transmission efficiency . . . 23
8.5 Transmission rate . . . 24
8.6 Plaque forming units . . . 25
8.7 All graphs together for scientific publication . . . 27
8.8 Other graphs for the publication . . . 29
8.8.1 Infection rate . . . 29
8.8.2 Dissemination efficiency . . . 31
8.8.3 Dissemination rate . . . 33
8.8.4 Transmission efficiency . . . 35
8.8.5 Transmission rate . . . 37
8.9 Plaque forming units . . . 39
8.10 Putting the graphs together . . . 40

**9 Session Information** **43**

# 1 Freezing Package versions

```
## (messages are omitted in this chunk)
##
library(checkpoint)
checkpoint("2022-05-04")
```

# 2 Load packages

```
## (warnings and messages are omitted from this chunk)
##
library(dplyr)
library(ggplot2)
library(binom)
library(ggpubr)
```

# 3 Getting data

We first get the data about infection, dissemination and transmission. Note that this data is aggregated at experiment-condition level. So, for example for the control group, in the fluctuating temperature at time point 7 days there is a value.

```
d.vectorCompetence <- readRDS("Data_and_models/data_vectorCompetence_aggregated_ready.RDS")
##
str(d.vectorCompetence)
```

```
tibble [8 x 12] (S3: tbl_df/tbl/data.frame)
 $ Time.fac                : Factor w/ 2 levels "7","10": 1 1 1 1 2 2 2 2
 $ ExperimentType          : Factor w/ 2 levels "Control","2nd Blood Meal": 1 1 2 2 1 1 2 2
 $ Temperature.fac         : Factor w/ 2 levels "FT","CT": 1 2 1 2 1 2 1 2
 $ infected.cases          : num [1:8] 21 26 18 6 29 21 21 9
 $ disseminated.cases      : num [1:8] 11 19 14 6 26 19 20 6
 $ transmitted.cases       : num [1:8] 1 11 6 1 3 7 2 2
 $ n.obs                   : int [1:8] 29 29 29 20 30 25 29 28
 $ infection.rate          : num [1:8] 0.724 0.897 0.621 0.3 0.967 ...
 $ dissemination.efficiency: num [1:8] 0.379 0.655 0.483 0.3 0.867 ...
 $ dissemination.rate      : num [1:8] 0.524 0.731 0.778 1 0.897 ...
 $ transmission.efficiency : num [1:8] 0.0345 0.3793 0.2069 0.05 0.1 ...
 $ transmission.rate       : num [1:8] 0.0909 0.5789 0.4286 0.1667 0.1154 ...
```

```
print(d.vectorCompetence, n = 3, width = Inf)
```

```
# A tibble: 8 x 12
  Time.fac ExperimentType Temperature.fac infected.cases disseminated.cases
  <fct>    <fct>          <fct>                    <dbl>              <dbl>
1 7        Control        FT                          21                 11
2 7        Control        CT                          26                 19
3 7        2nd Blood Meal FT                          18                 14
  transmitted.cases n.obs infection.rate dissemination.efficiency
              <dbl> <int>          <dbl>                    <dbl>
1                 1    29          0.724                    0.379
2                11    29          0.897                    0.655
3                 6    29          0.621                    0.483
```

```
  dissemination.rate transmission.efficiency transmission.rate
               <dbl>                   <dbl>             <dbl>
1              0.524                  0.0345            0.0909
2              0.731                  0.379             0.579
3              0.778                  0.207             0.429
# ... with 5 more rows
```

We then get the data about PFU results. Note that this data has not been aggregated, but is rather measured at mosquito level.

```
d.PFU <- readRDS("Data_and_models/data_PFU_ready.RDS")
##
str(d.PFU)
```

```
tibble [219 x 5] (S3: tbl_df/tbl/data.frame)
 $ Time           : num [1:219] 7 7 7 7 7 7 7 7 7 7 ...
 $ Time.fac       : Factor w/ 2 levels "7","10": 1 1 1 1 1 1 1 1 1 1 ...
 $ Temperature.fac: Factor w/ 2 levels "FT","CT": 2 2 2 2 2 2 2 2 2 2 ...
 $ ExperimentType : Factor w/ 2 levels "Control","2nd Blood Meal": 1 1 1 1 1 1 1 1 1 1 ...
 $ PFU.num        : num [1:219] 0 50 200 0 10 0 0 0 10 0 ...
```

```
print(d.PFU, n = 3, width = Inf)
```

```
# A tibble: 219 x 5
   Time Time.fac Temperature.fac ExperimentType PFU.num
  <dbl> <fct>    <fct>           <fct>            <dbl>
1     7 7        CT              Control              0
2     7 7        CT              Control             50
3     7 7        CT              Control            200
# ... with 216 more rows
```

# 4 Estimating confidence intervals for binomial proportions

Let's compute the confidence intervals for the proportions measured in the lab. Note that we use the Wilson method to estimate confidence intervals.

```
## 1) Infection rate
d.ci.binom.infection.rate <- binom.confint(
  x = d.vectorCompetence$infected.cases,
  n = d.vectorCompetence$n.obs,
  methods = "wilson")
# d.ci.binom.infection.rate
##
d.vectorCompetence$lower_infection.rate <- d.ci.binom.infection.rate$lower
d.vectorCompetence$upper_infection.rate <- d.ci.binom.infection.rate$upper
# print(d.vectorCompetence, width = Inf)
##
##
## 2) Dissemination efficiency
d.ci.binom.dissemination.efficiency <- binom.confint(
  x = d.vectorCompetence$disseminated.cases,
  n = d.vectorCompetence$n.obs,
  methods = "wilson")
##
d.vectorCompetence$lower_dissemination.efficiency <- d.ci.binom.dissemination.efficiency$lower
```

```r
d.vectorCompetence$upper_dissemination.efficiency <- d.ci.binom.dissemination.efficiency$upper
##
##
## 3) Dissemination rate
d.ci.binom.dissemination.rate <- binom.confint(
  x = d.vectorCompetence$disseminated.cases,
  n = d.vectorCompetence$infected.cases,
  methods = "wilson")
##
d.vectorCompetence$lower_dissemination.rate <- d.ci.binom.dissemination.rate$lower
d.vectorCompetence$upper_dissemination.rate <- d.ci.binom.dissemination.rate$upper
##
##
## 4) Transmission efficiency
d.ci.binom.transmission.efficiency <- binom.confint(
  x = d.vectorCompetence$transmitted.cases,
  n = d.vectorCompetence$n.obs,
  methods = "wilson")
##
d.vectorCompetence$lower_transmission.efficiency <- d.ci.binom.transmission.efficiency$lower
d.vectorCompetence$upper_transmission.efficiency <- d.ci.binom.transmission.efficiency$upper
##
##
## 5) Transmission rate
d.ci.binom.transmission.rate <- binom.confint(
  x = d.vectorCompetence$transmitted.cases,
  n = d.vectorCompetence$disseminated.cases,
  methods = "wilson")
##
d.vectorCompetence$lower_transmission.rate <- d.ci.binom.transmission.rate$lower
d.vectorCompetence$upper_transmission.rate <- d.ci.binom.transmission.rate$upper
##
# print(d.vectorCompetence, width = Inf)
```

Let's check one combination.

```r
## check
## 1) Infection
d.ci.binom.infection.rate[1, ]
```

```
  method  x  n mean lower upper
1 wilson 21 29 0.72  0.54  0.85
```

```r
d.vectorCompetence[1, ] %>%
  select(contains("infection")) %>%
  print(width = Inf)
```

```
# A tibble: 1 x 3
  infection.rate lower_infection.rate upper_infection.rate
           <dbl>                <dbl>                <dbl>
1          0.724                0.543                0.853
```

```r
##
## 2) Dissemination efficiency
d.ci.binom.dissemination.efficiency[1, ]
```

```
  method  x  n mean lower upper
1 wilson 11 29 0.38  0.23  0.56
```

```
d.vectorCompetence[1, ] %>%
  select(contains("dissemination.efficiency")) %>%
  print(width = Inf)
```

```
# A tibble: 1 x 3
  dissemination.efficiency lower_dissemination.efficiency
                     <dbl>                          <dbl>
1                    0.379                          0.227
  upper_dissemination.efficiency
                           <dbl>
1                          0.560
```

```
##
## 3) Dissemination rate
d.ci.binom.dissemination.rate[1, ]
```

```
  method  x  n mean lower upper
1 wilson 11 21 0.52  0.32  0.72
```

```
d.vectorCompetence[1, ] %>%
  select(contains("dissemination.rate")) %>%
  print(width = Inf)
```

```
# A tibble: 1 x 3
  dissemination.rate lower_dissemination.rate upper_dissemination.rate
               <dbl>                    <dbl>                    <dbl>
1              0.524                    0.324                    0.717
```

```
##
## 4) Transmission efficiency
d.ci.binom.transmission.efficiency[1, ]
```

```
  method x  n  mean  lower upper
1 wilson 1 29 0.034 0.0061  0.17
```

```
d.vectorCompetence[1, ] %>%
  select(contains("transmission.efficiency")) %>%
  print(width = Inf)
```

```
# A tibble: 1 x 3
  transmission.efficiency lower_transmission.efficiency
                    <dbl>                         <dbl>
1                  0.0345                       0.00611
  upper_transmission.efficiency
                          <dbl>
1                         0.172
```

```
##
## 5) Transmission rate
d.ci.binom.transmission.rate[1, ]
```

```
  method x  n  mean lower upper
1 wilson 1 11 0.091 0.016  0.38
```

```
d.vectorCompetence[1, ] %>%
  select(contains("transmission.rate")) %>%
```

```
  print(width = Inf)
```

```
# A tibble: 1 x 3
  transmission.rate lower_transmission.rate upper_transmission.rate
              <dbl>                   <dbl>                   <dbl>
1            0.0909                  0.0162                   0.377
```

```
##
## they all makes sense (for this one case)
```

We store the results in a spreadsheet.

```
write.csv(d.vectorCompetence,
          file = "Data_and_models/vectorCompetence_with_CIs.csv",
          row.names = FALSE)
```

# 5 Graphical Analysis

## 5.1 Proto-plot

Many graphs will have the same building blocks. Therefore, we start by creating a proto-plot.

```
gg.proto <- ggplot(
  data = d.vectorCompetence,
  mapping = aes(x = Time.fac,
                colour = ExperimentType)) +
  scale_y_continuous(limits = c(0,1), minor_breaks = NULL) +
  geom_hline(yintercept = c(0,1), colour = "gray") +
  geom_point(position = position_dodge(0.25)) +
  geom_linerange(position = position_dodge(0.25)) +
  facet_grid(.~Temperature.fac) +
  theme_bw()
```

## 5.2 Infection rate

```
gg.infection.rate <- gg.proto +
  aes(y = infection.rate,
      ymin = lower_infection.rate, ymax = upper_infection.rate)
##
gg.infection.rate
```

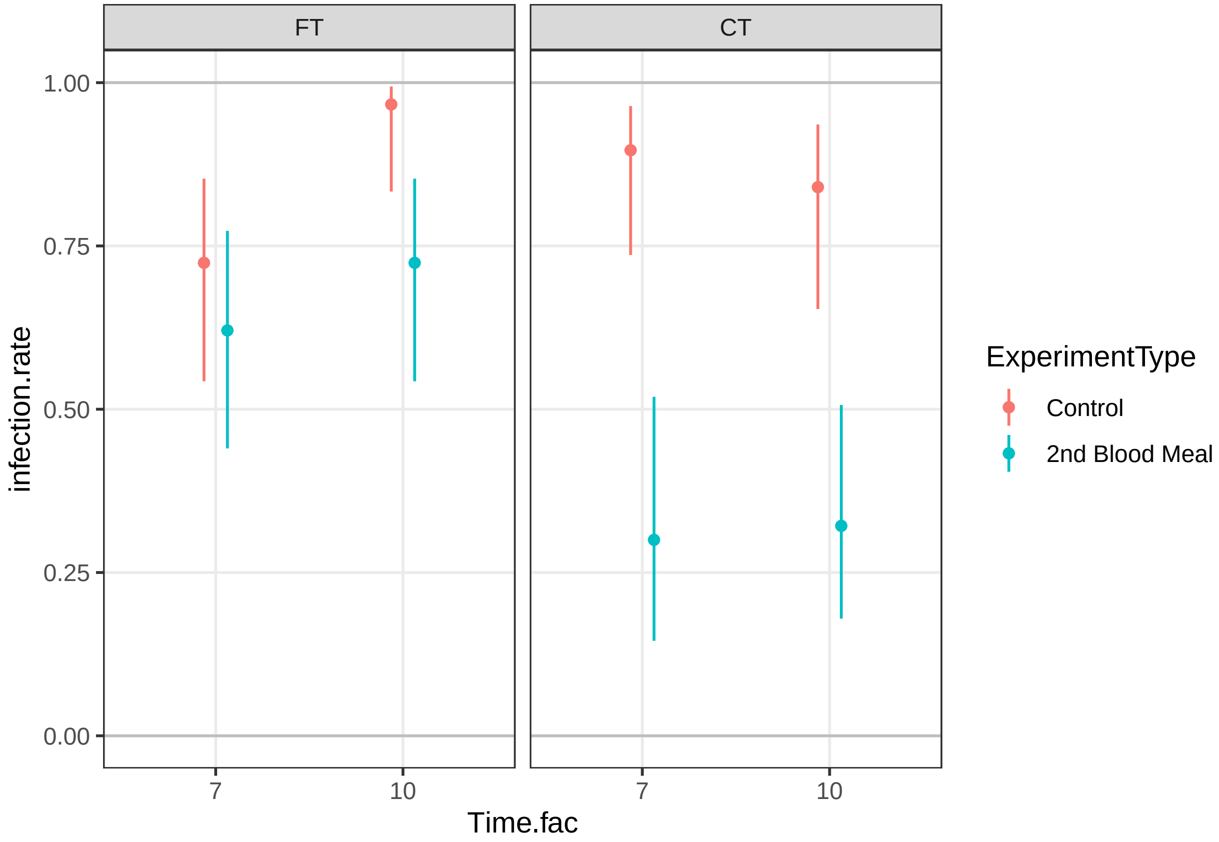


### 5.3 Dissemination efficiency

```
gg.dissemination.efficiency <- gg.proto +
  aes(y = dissemination.efficiency,
      ymin = lower_dissemination.efficiency,
      ymax = upper_dissemination.efficiency)
##
gg.dissemination.efficiency
```

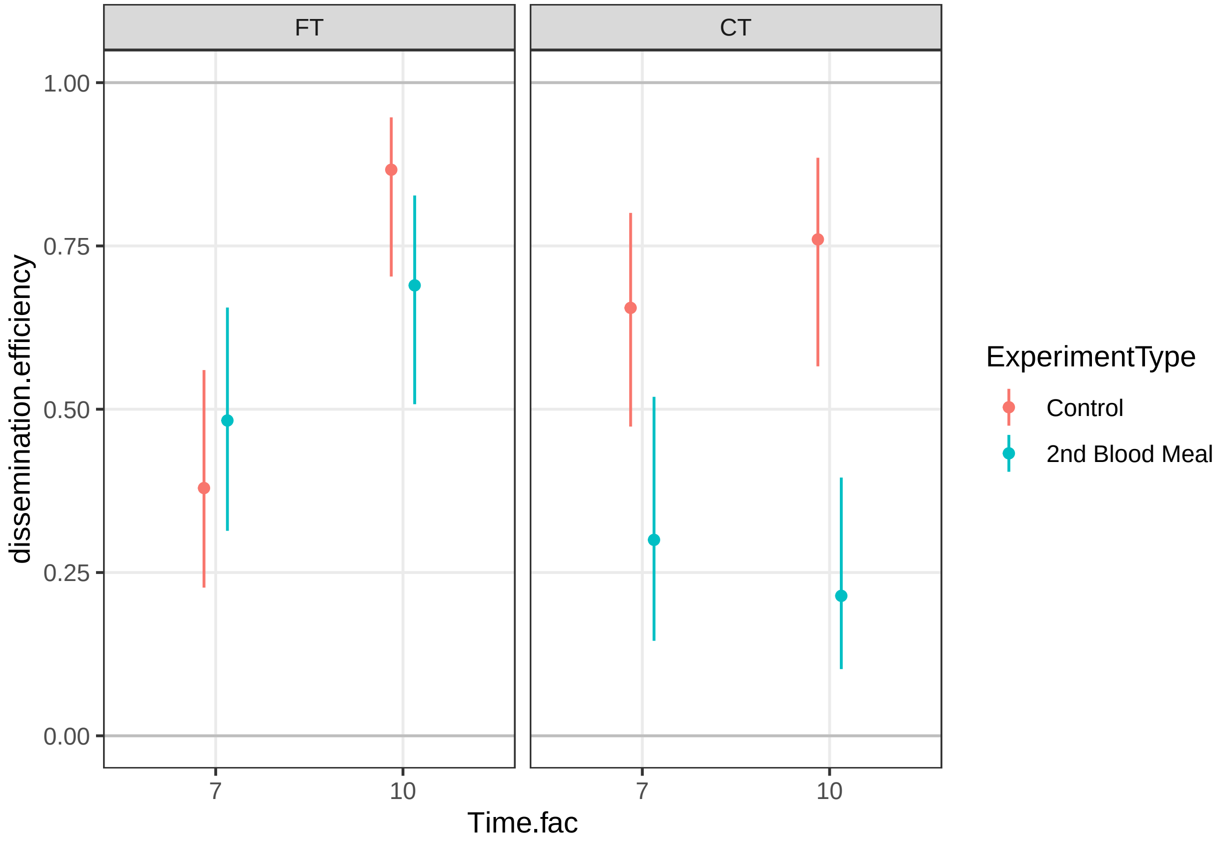


### 5.4 Dissemination rate

```
gg.dissemination.rate <- gg.proto +
  aes(y = dissemination.rate,
      ymin = lower_dissemination.rate,
      ymax = upper_dissemination.rate)
##
gg.dissemination.rate
```

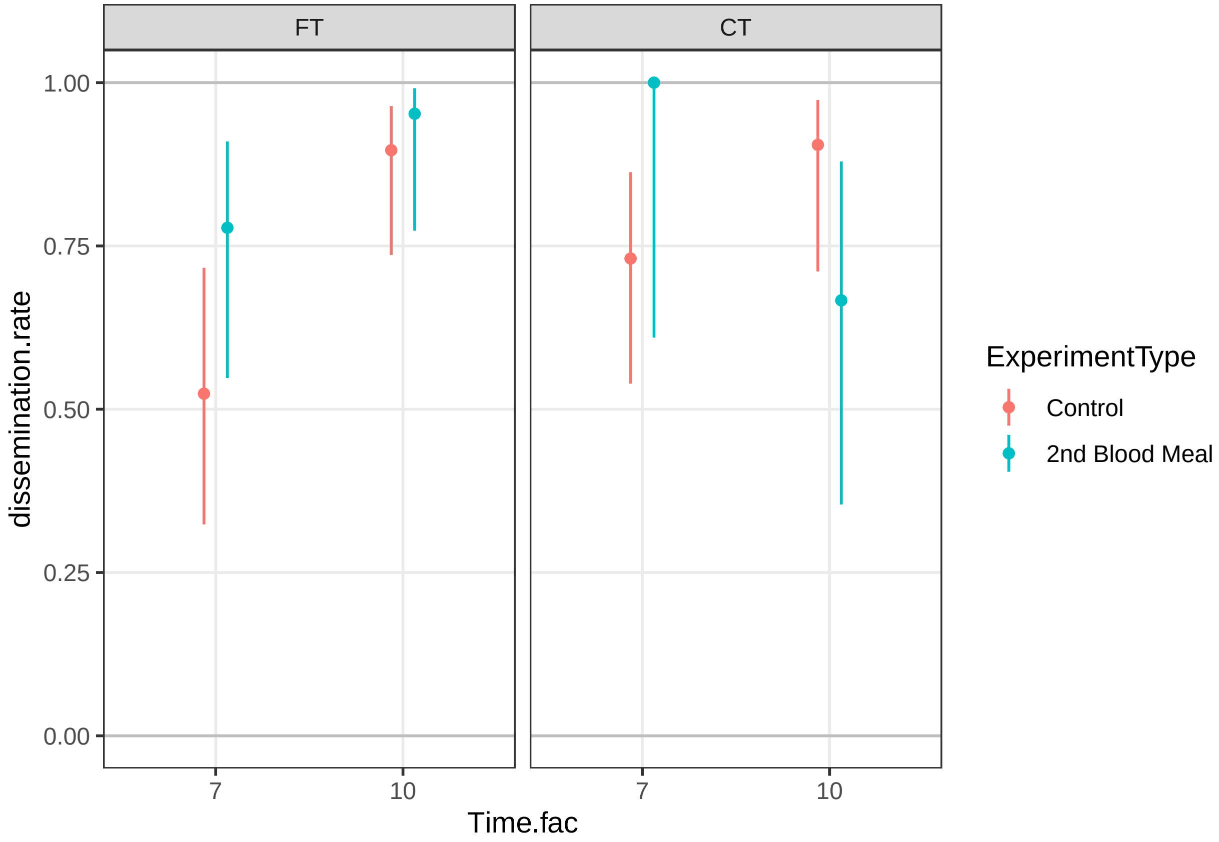


### 5.5 Transmission efficiency

```
gg.trasmission.efficiency <- gg.proto +
  aes(y = transmission.efficiency,
      ymin = lower_transmission.efficiency,
      ymax = upper_transmission.efficiency)
##
gg.trasmission.efficiency
```

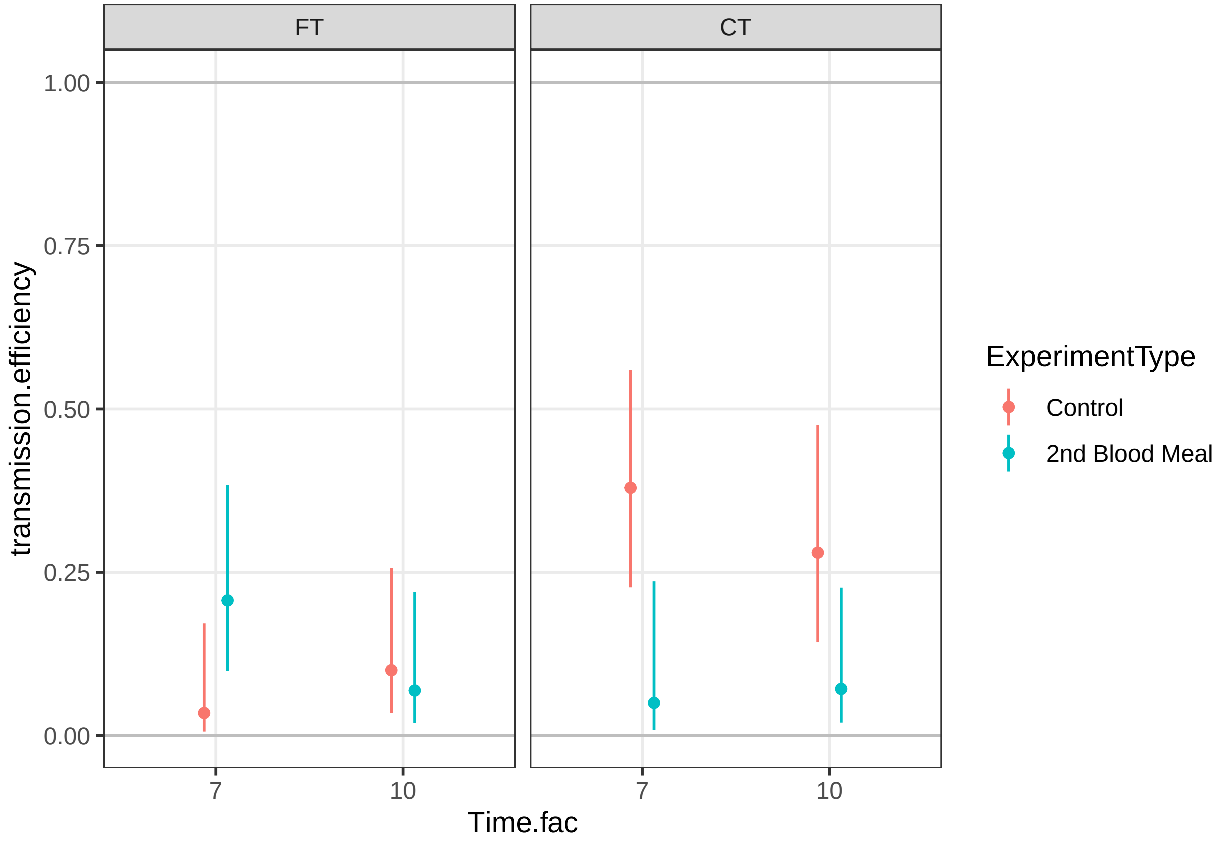


### 5.6 Transmission rate

```
gg.trasmission.rate <- gg.proto +
  aes(y = transmission.rate,
      ymin = lower_transmission.rate,
      ymax = upper_transmission.rate)
##
gg.trasmission.rate
```

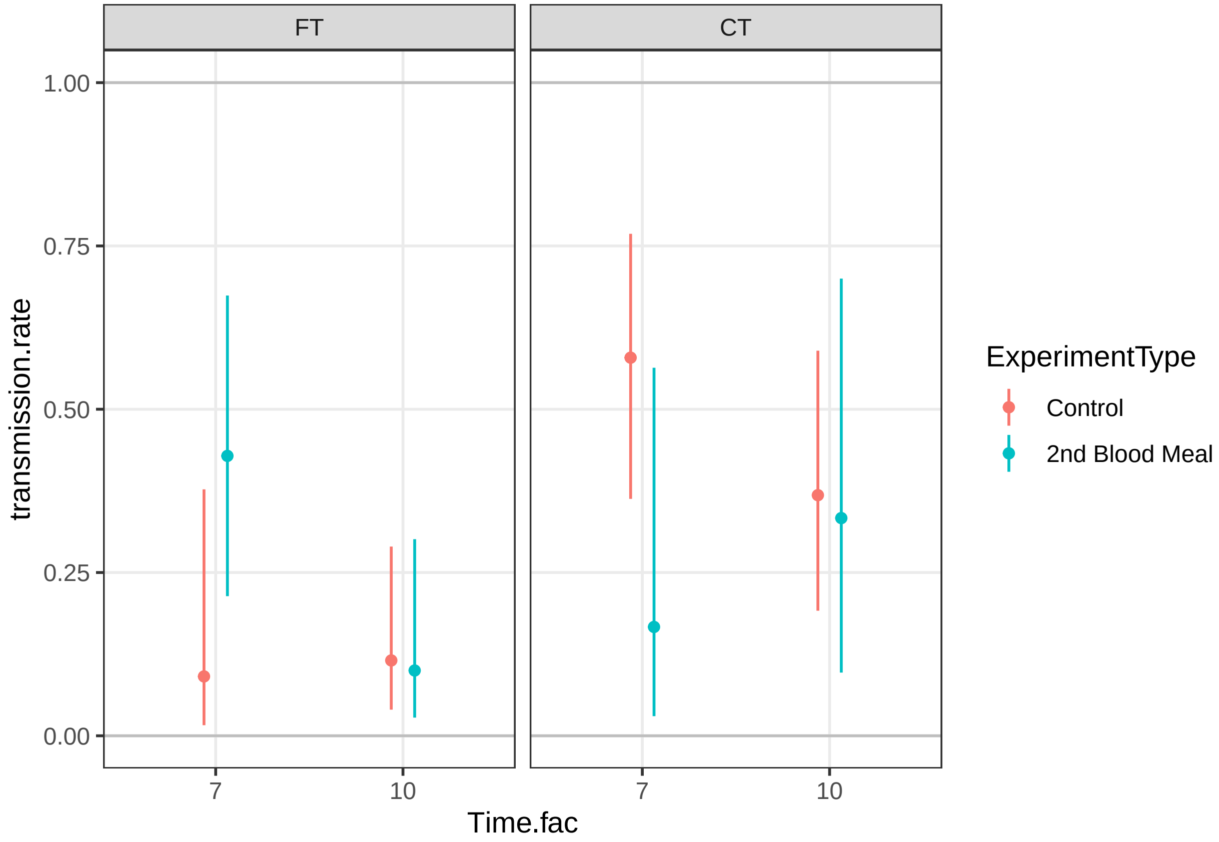


## 5.7 PFU

This variable is visualised on the mosquito level. Note that the y-axis is square-root transformed as we are dealing with count data.

```
gg.PFU <- ggplot(data = d.PFU,
       mapping = aes(y = PFU.num,
                     x = Time,
                     colour = ExperimentType)) +
  scale_y_sqrt() +
  geom_hline(yintercept = 0, col = "gray") +
  geom_jitter(alpha = 0.2, width = 0.1, height = 0.1) +
  facet_grid(~Temperature.fac) +
  stat_summary(fun = mean,
               geom = "line") +
  theme_bw()
##
gg.PFU
```

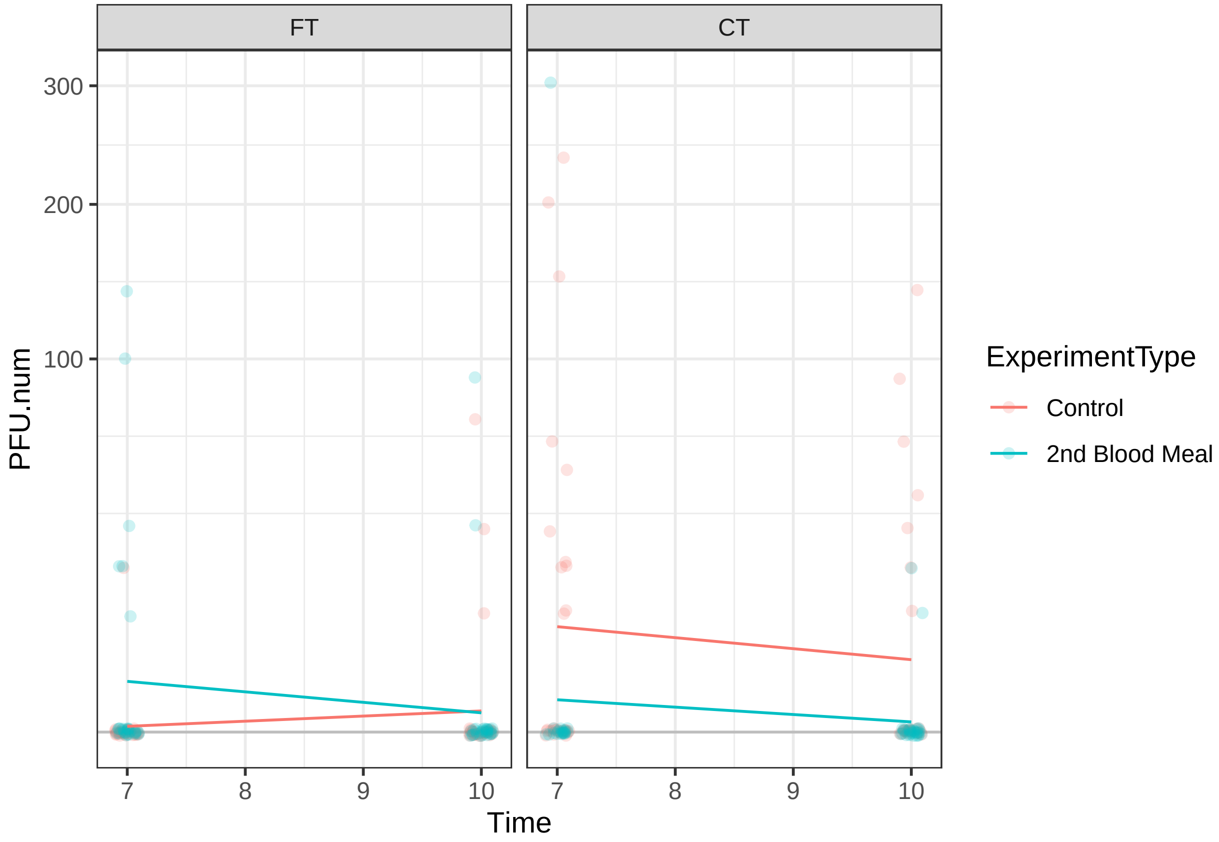


These results are consistent with the ones of infection, dissemination and transmission. In particular controls seems to have higher values at constant temperature, while there is no systematic difference at fluctuating temperature.

In three out of four cases PFU decreases over time.

## 5.8 All graphs together

```
ggarrange(gg.infection.rate,
          gg.dissemination.efficiency,
          gg.dissemination.rate,
          gg.trasmission.efficiency,
          gg.trasmission.rate,
          gg.PFU,
          common.legend = TRUE)
```

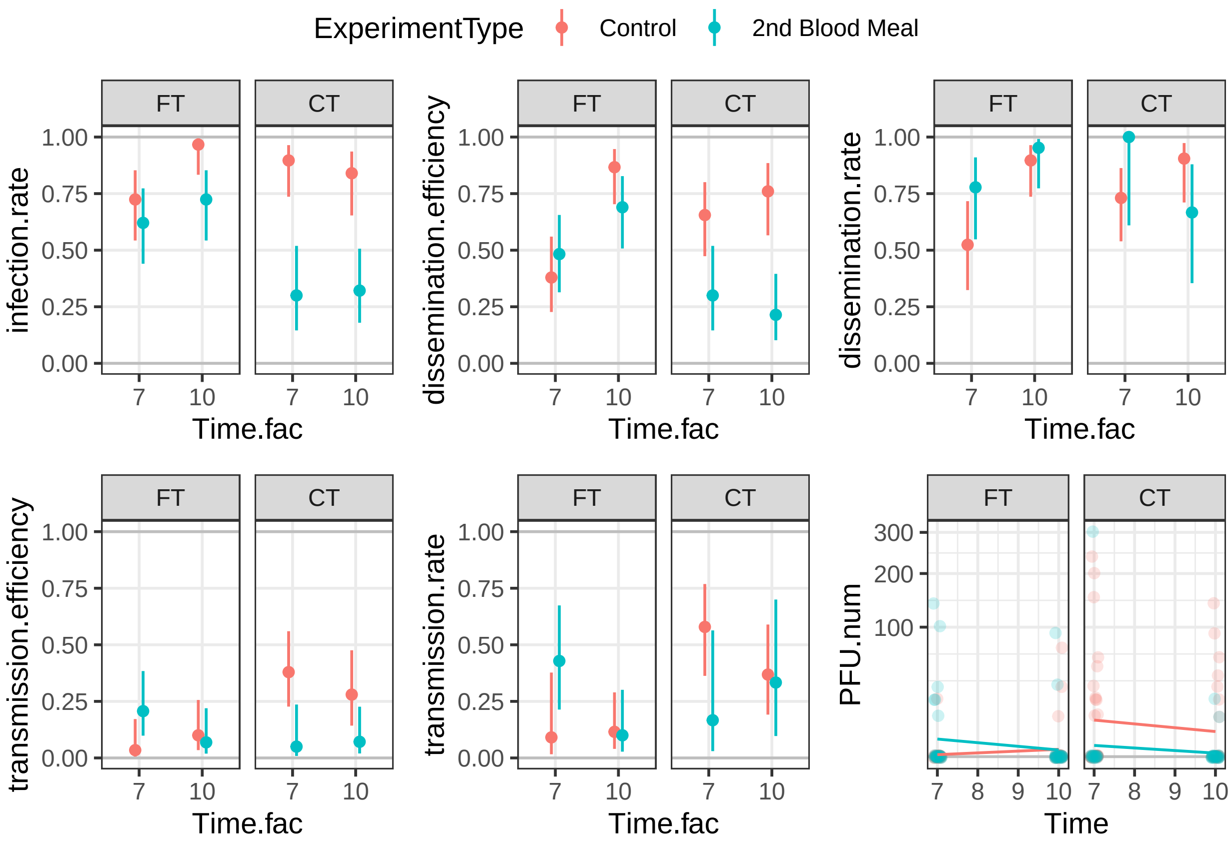


In general:

- “2nd Blood Meal” are consistently lower rates/efficiencies in constant conditions (11 out of 12 cases)
- this difference is not present in fluctuating conditions
- there seems to be no consistent time effect
- the temperature seem to play a role in the sense that the experiment type effect is larger in constant conditions (which implies that an interaction is present)

# 6 PFU (formal modelling)

We model *PFU.num*. Note that this model is not fitted on the aggregated data, but rather on the observation-level dataset. Note also that we use the "quasipoisson" (quasi)-family to account for the non constant variance of the response variable (count data).

```
glm.PFU_start <- glm(
  PFU.num ~ Time.fac * Temperature.fac * ExperimentType,
  family = "quasipoisson",
  data = d.PFU)
summary(glm.PFU_start)
```

```

Call:
glm(formula = PFU.num ~ Time.fac * Temperature.fac * ExperimentType,
    family = "quasipoisson", data = d.PFU)

Deviance Residuals:
   Min      1Q  Median      3Q     Max
 -7.47   -5.09   -2.71   -1.32   35.03

Coefficients:
                                                          Estimate Std. Error
(Intercept)                                                 -0.372      2.087
Time.fac10                                                   1.671      2.269
Temperature.facCT                                            3.701      2.112
ExperimentType2nd Blood Meal                                 2.773      2.151
Time.fac10:Temperature.facCT                                -2.253      2.340
Time.fac10:ExperimentType2nd Blood Meal                     -2.652      2.479
Temperature.facCT:ExperimentType2nd Blood Meal              -3.394      2.242
Time.fac10:Temperature.facCT:ExperimentType2nd Blood Meal    0.595      3.109
                                                          t value Pr(>|t|)
(Intercept)                                                 -0.18    0.859
Time.fac10                                                   0.74    0.462
Temperature.facCT                                            1.75    0.081 .
ExperimentType2nd Blood Meal                                 1.29    0.199
Time.fac10:Temperature.facCT                                -0.96    0.337
Time.fac10:ExperimentType2nd Blood Meal                     -1.07    0.286
Temperature.facCT:ExperimentType2nd Blood Meal              -1.51    0.131
Time.fac10:Temperature.facCT:ExperimentType2nd Blood Meal    0.19    0.848
---
Signif. codes:  0 '***' 0.001 '**' 0.01 '*' 0.05 '.' 0.1 ' ' 1

(Dispersion parameter for quasipoisson family taken to be 87)

    Null deviance: 10008.2  on 218  degrees of freedom
Residual deviance:  8188.3  on 211  degrees of freedom
AIC: NA

Number of Fisher Scoring iterations: 8
```

Let's simplify the model. We consider the 5% threshold for model simplification.

```
drop1(glm.PFU_start, test = "Chisq")
```

```
Single term deletions

Model:
PFU.num ~ Time.fac * Temperature.fac * ExperimentType
                                       Df Deviance scaled dev. Pr(>Chi)
<none>                                        8188
Time.fac:Temperature.fac:ExperimentType  1     8192      0.0372     0.85
```

We drop the 3-fold interaction from the model.

```r
glm.PFU_all2fold <- update(glm.PFU_start,
                                   . ~ . - Time.fac : Temperature.fac : ExperimentType)
##
# summary(glm.PFU_all2fold)
```

Let's perform inference.

```r
drop1(glm.PFU_all2fold, test = "Chisq")
```

```
Single term deletions

Model:
PFU.num ~ Time.fac + Temperature.fac + ExperimentType + Time.fac:Temperature.fac +
    Time.fac:ExperimentType + Temperature.fac:ExperimentType
                              Df Deviance scaled dev. Pr(>Chi)
<none>                              8192
Time.fac:Temperature.fac        1     8372        2.09   0.1480
Time.fac:ExperimentType         1     8481        3.36   0.0669 .
Temperature.fac:ExperimentType  1     8787        6.91   0.0086 **
---
Signif. codes:  0 '***' 0.001 '**' 0.01 '*' 0.05 '.' 0.1 ' ' 1
```

We drop the "Time.fac:Temperature.fac" interaction and perform inference again.

```r
glm.PFU_two2fold <- update(glm.PFU_all2fold,
                                   . ~ . - Time.fac : Temperature.fac)
##
# summary(glm.PFU_two2fold)
```

Let's perform inference.

```r
drop1(glm.PFU_two2fold, test = "Chisq")
```

```
Single term deletions

Model:
PFU.num ~ Time.fac + Temperature.fac + ExperimentType + Time.fac:ExperimentType +
    Temperature.fac:ExperimentType
                              Df Deviance scaled dev. Pr(>Chi)
<none>                              8372
Time.fac:ExperimentType         1     8508        1.36    0.244
Temperature.fac:ExperimentType  1     8821        4.47    0.034 *
---
Signif. codes:  0 '***' 0.001 '**' 0.01 '*' 0.05 '.' 0.1 ' ' 1
```

We drop the "Time.fac:ExperimentType" interaction and perform inference again.

```r
glm.PFU_one2fold <- update(glm.PFU_two2fold,
                                   . ~ . - Time.fac : ExperimentType)
##
# summary(glm.PFU_one2fold)
```

Let's perform inference.

```r
drop1(glm.PFU_one2fold, test = "Chisq")
```

```
Single term deletions

Model:
PFU.num ~ Time.fac + Temperature.fac + ExperimentType + Temperature.fac:ExperimentType
                               Df Deviance scaled dev. Pr(>Chi)
<none>                               8508
Time.fac                        1     8799         2.7    0.100
Temperature.fac:ExperimentType  1     8972         4.3    0.038 *
---
Signif. codes:  0 '***' 0.001 '**' 0.01 '*' 0.05 '.' 0.1 ' ' 1
```

So, the final "PFU" model is:

```r
summary(glm.PFU_one2fold)
```

```

Call:
glm(formula = PFU.num ~ Time.fac + Temperature.fac + ExperimentType +
    Temperature.fac:ExperimentType, family = "quasipoisson",
    data = d.PFU)

Deviance Residuals:
   Min      1Q  Median      3Q     Max
 -7.70   -4.56   -3.04   -1.78   38.19

Coefficients:
                                              Estimate Std. Error t value
(Intercept)                                      1.111      0.925    1.20
Time.fac10                                      -0.778      0.492   -1.58
Temperature.facCT                                2.278      0.959    2.37
ExperimentType2nd Blood Meal                     1.230      1.037    1.19
Temperature.facCT:ExperimentType2nd Blood Meal  -2.312      1.223   -1.89
                                              Pr(>|t|)
(Intercept)                                      0.231
Time.fac10                                       0.115
Temperature.facCT                                0.018 *
ExperimentType2nd Blood Meal                     0.237
Temperature.facCT:ExperimentType2nd Blood Meal   0.060 .
---
Signif. codes:  0 '***' 0.001 '**' 0.01 '*' 0.05 '.' 0.1 ' ' 1

(Dispersion parameter for quasipoisson family taken to be 108)

    Null deviance: 10008.2  on 218  degrees of freedom
Residual deviance:  8508.2  on 214  degrees of freedom
AIC: NA
```

```
Number of Fisher Scoring iterations: 8
```

These results are concordant with those of the graphical analysis.

### 6.1 PFU (visualising model fit)

To visualise the model fit we must extract the fitted values and the associated standard errors.

```
d.PFU$fit.PFU <- fitted(glm.PFU_one2fold) ## counts space
##
d.fit.PFU_LP <- predict(glm.PFU_one2fold,
                             se.fit = TRUE) ## LP space
d.PFU$lower.PFU <- exp(
  d.fit.PFU_LP$fit -
    1.96 * d.fit.PFU_LP$se.fit)
d.PFU$upper.PFU <- exp(
  d.fit.PFU_LP$fit +
    1.96 * d.fit.PFU_LP$se.fit)
```

We visualise the model fit for *PFU*.

```
ggplot(data = d.PFU,
       mapping = aes(y = fit.PFU,
                     x = Time,
                     ymin = lower.PFU,
                     ymax = upper.PFU,
                     colour = ExperimentType)) +
  scale_y_sqrt() +
  geom_hline(yintercept = 0, col = "gray") +
  geom_point(position = position_dodge(0.25)) +
  geom_line() +
  geom_linerange(position = position_dodge(0.25)) +
  facet_grid(~Temperature.fac) +
  theme_bw()
```

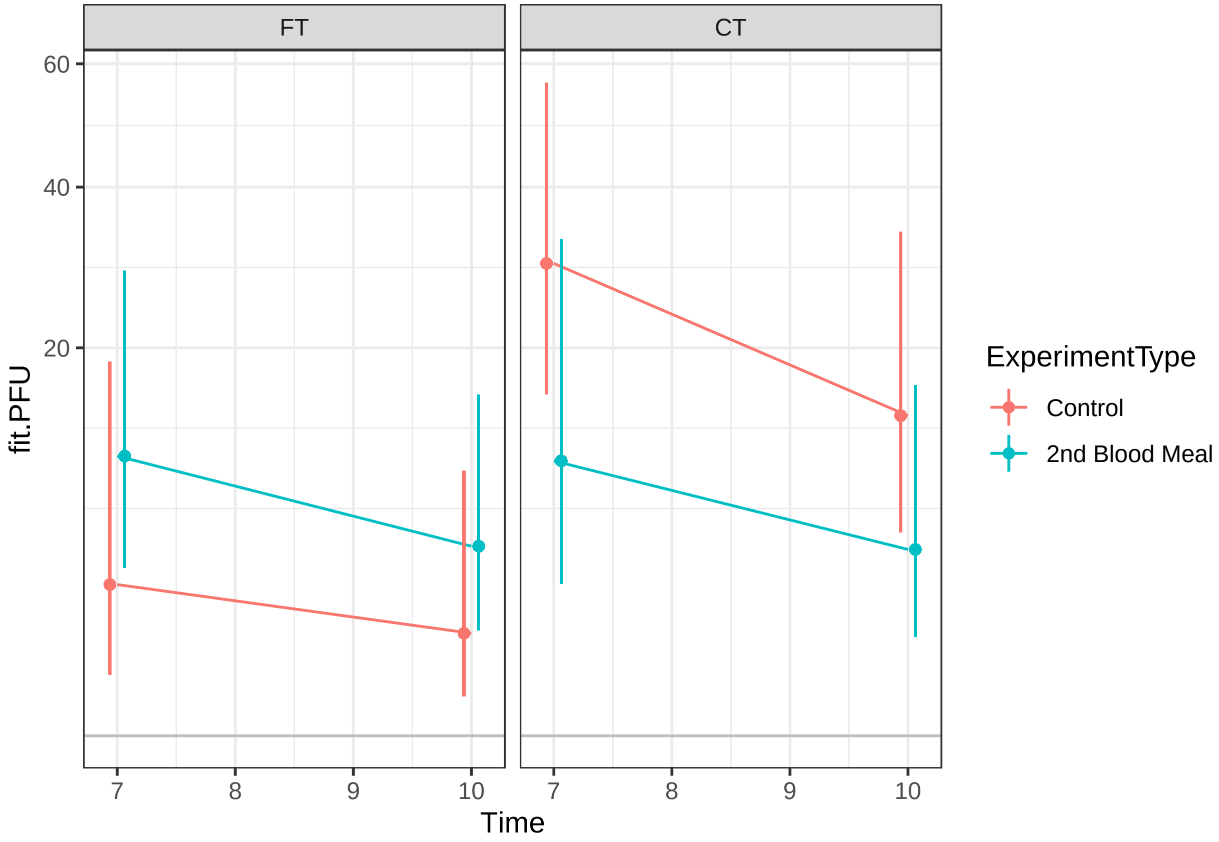


Note, that the confidence intervals reported here refer to the predicted values not the model-coefficients themselves.

# 7 Methods description

All rates and efficiencies computed for infection, dissemination and transformation are analysed as binomial data. The Wilson method was used to estimate 95% confidence intervals for the binomial proportions.

The plaque-forming unit data was analysed with a Generalised Linear Model with family set to quasi-poisson to account for overdispersion. The starting model contained the three-fold interaction among days post-infection, temperature regime (constant or fluctuating) and experiment type (control or 2nd blood meal). The significance level was set at 5%.

For details about the statistical analyses refer to the appendix.

# 8 Figures for scientific publication

For the publication the x-axis is renamed to "dpi" (ie. days post-infection).

```
gg.proto_sp <- gg.proto +
  xlab(label = "dpi")
## the suffix "_sp" stands for "scientific publication"
```

## 8.1 Infection rate

```r
gg.infection.rate_sp <- gg.proto_sp +
  aes(y = infection.rate,
      ymin = lower_infection.rate,
      ymax = upper_infection.rate) +
  ylab(label = "infection rate")
##
gg.infection.rate_sp
```

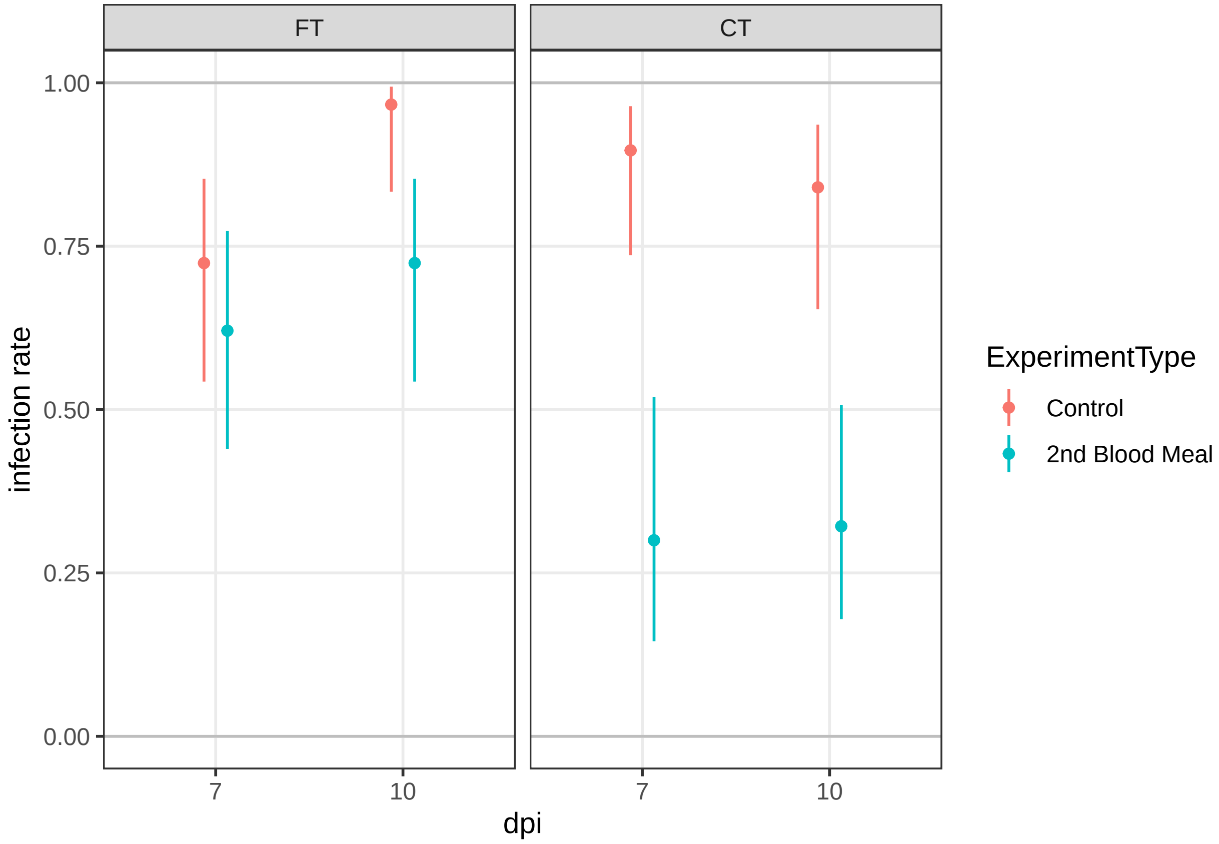


## 8.2 Dissemination efficiency

```r
gg.dissemination.efficiency_sp <- gg.proto_sp +
  aes(y = dissemination.efficiency,
      ymin = lower_dissemination.efficiency,
      ymax = upper_dissemination.efficiency) +
  ylab(label = "dissemination efficiency")
##
gg.dissemination.efficiency_sp
```

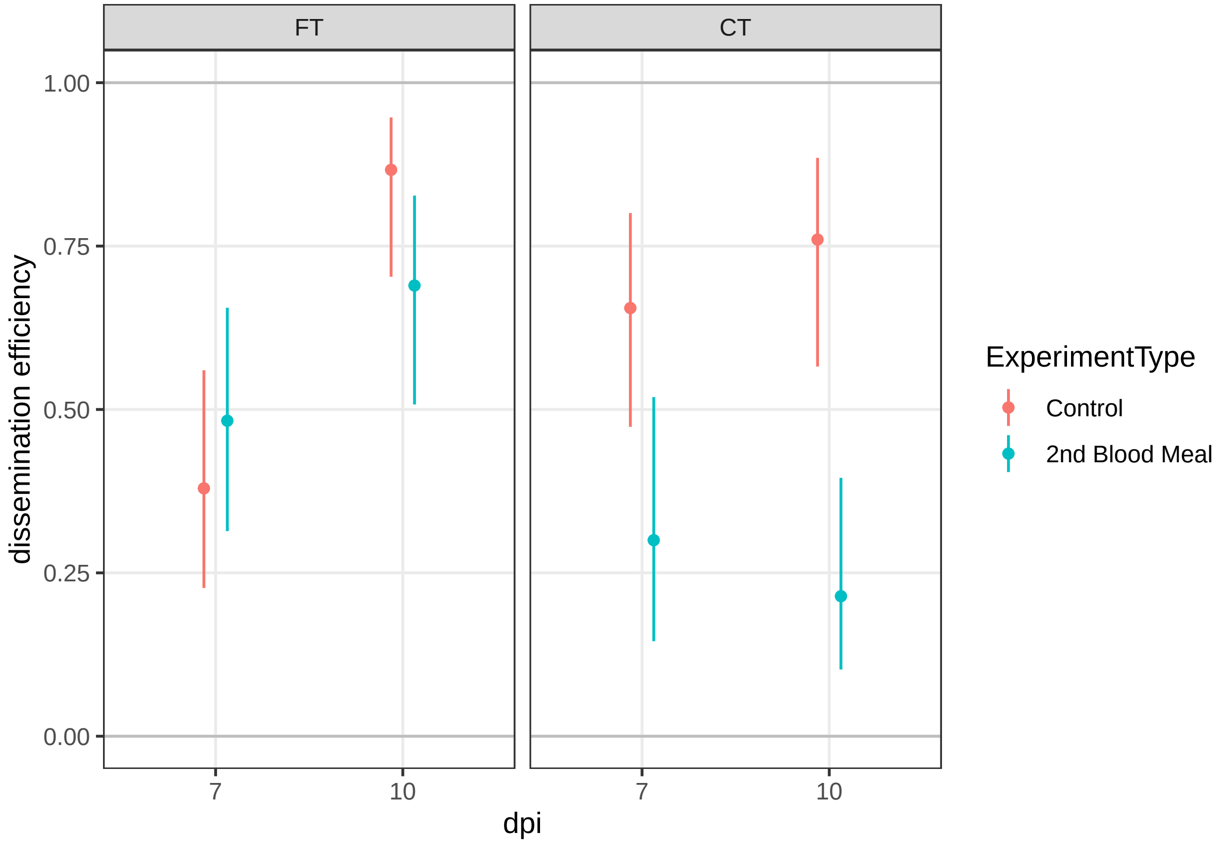


### 8.3 Dissemination rate

```
gg.dissemination.rate_sp <- gg.proto_sp +
  aes(y = dissemination.rate,
      ymin = lower_dissemination.rate,
      ymax = upper_dissemination.rate) +
  ylab(label = "dissemination rate")
##
gg.dissemination.rate_sp
```

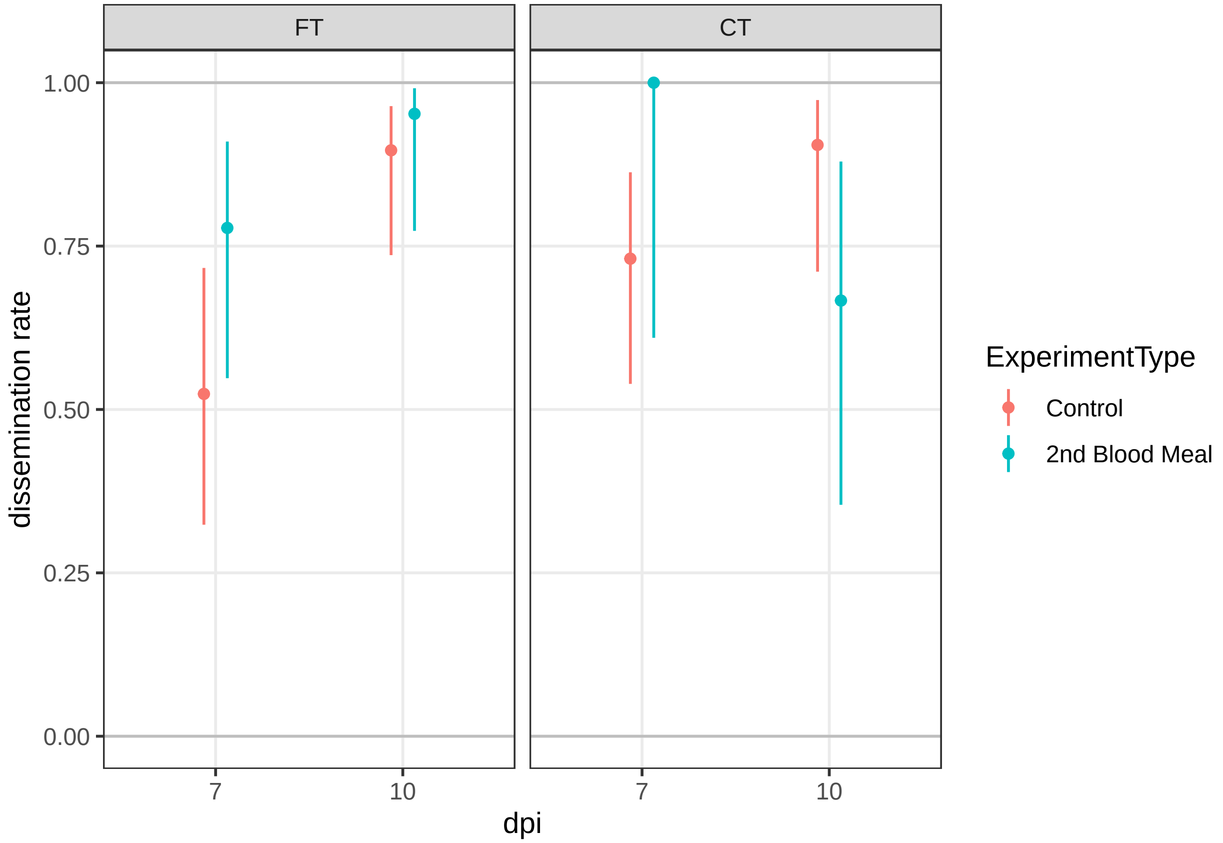


## 8.4 Transmission efficiency

```
gg.trasmission.efficiency_sp <- gg.proto_sp +
  aes(y = transmission.efficiency,
      ymin = lower_transmission.efficiency,
      ymax = upper_transmission.efficiency) +
  ylab(label = "transmission efficiency")
##
gg.trasmission.efficiency_sp
```

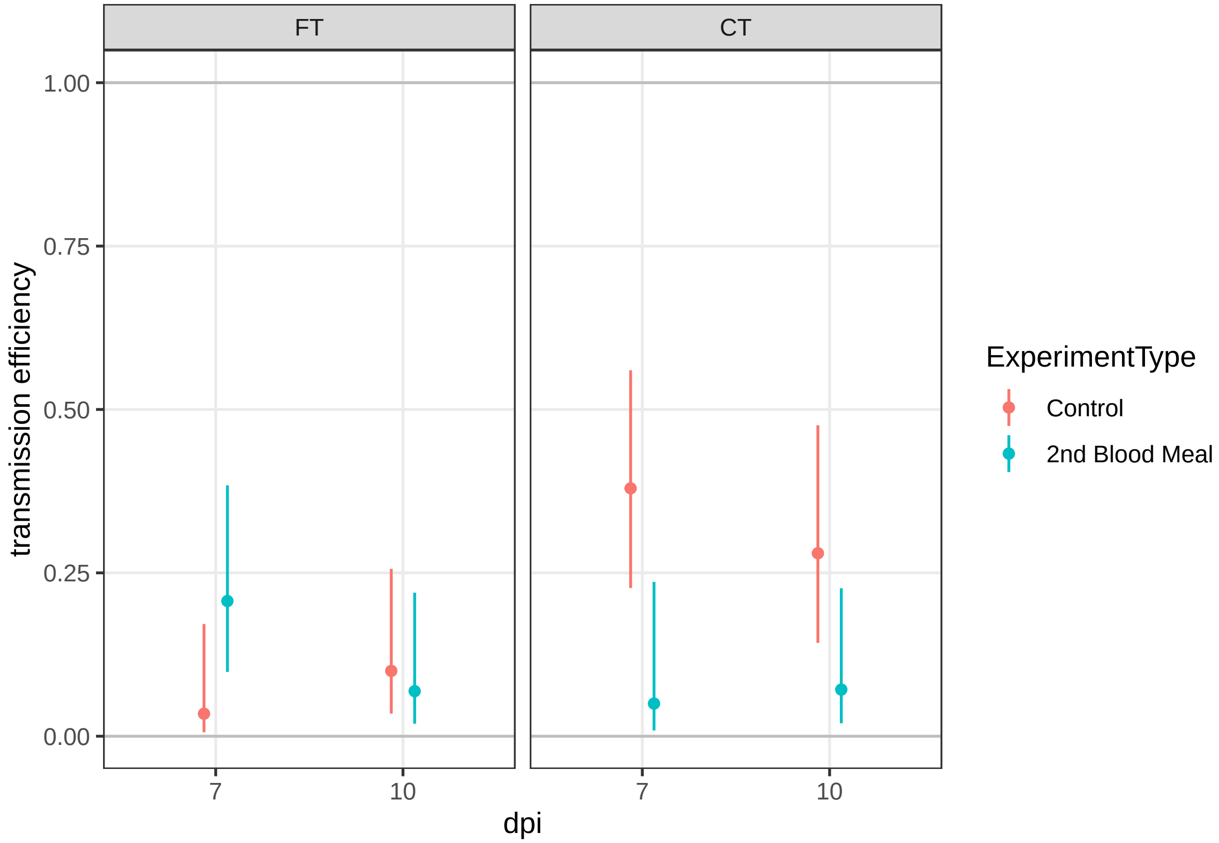


### 8.5 Transmission rate

```
gg.trasmission.rate_sp <- gg.proto_sp +
  aes(y = transmission.rate,
      ymin = lower_transmission.rate,
      ymax = upper_transmission.rate) +
  ylab(label = "transmission rate")
##
gg.trasmission.rate_sp
```

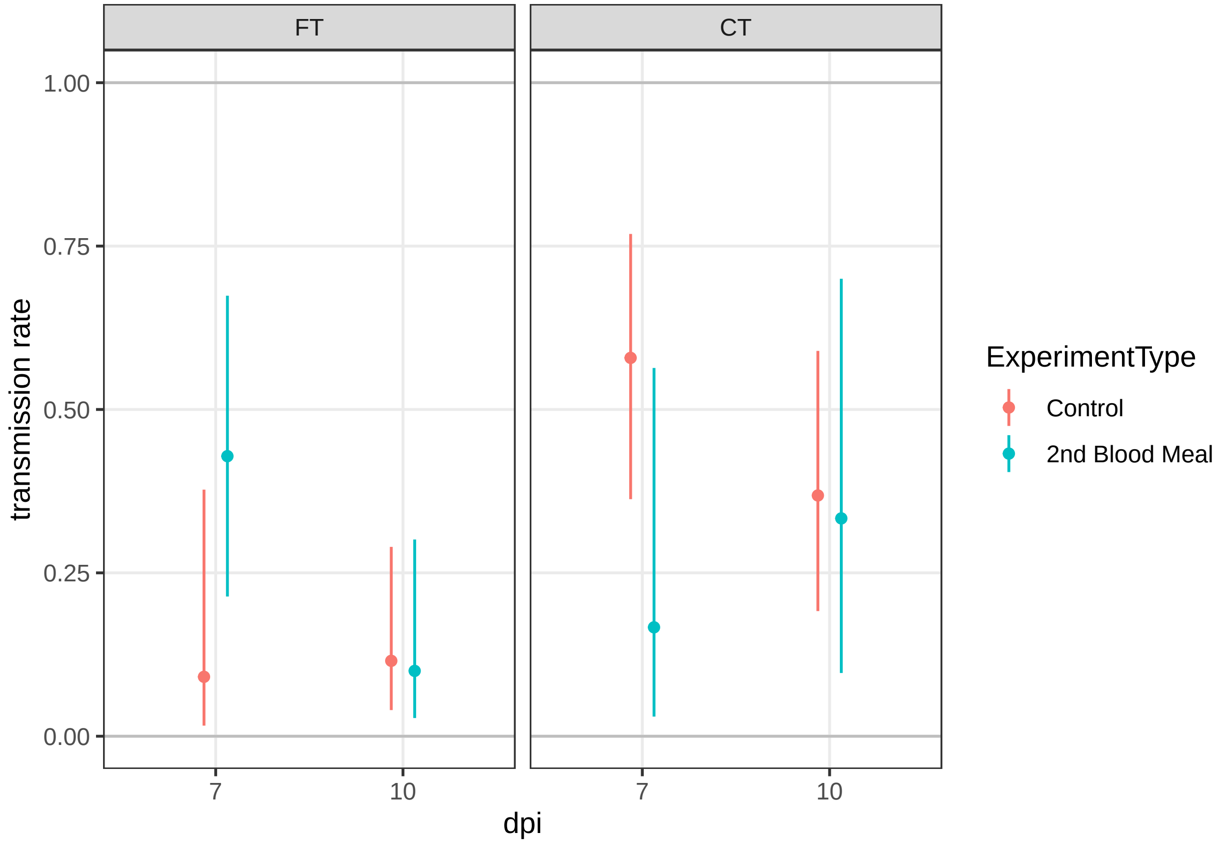


## 8.6 Plaque forming units

```
gg.PFU_sp <- gg.PFU +
  xlab(label = "dpi") +
  ylab("PFU / female")
##
gg.PFU_sp
```

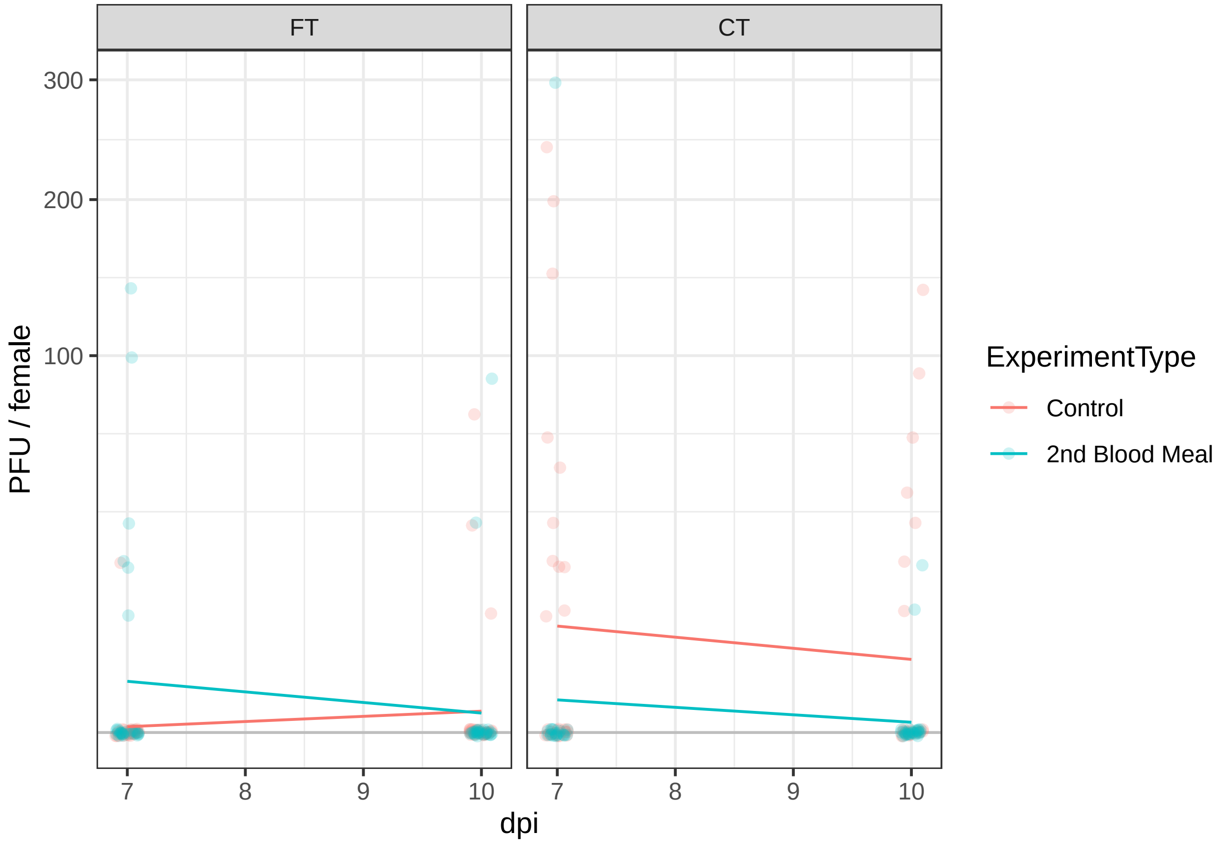


On this graph the number of plaque-forming units per female is visualised against days post-infection (ie. "dpi"). Note, that the y-axis has been square-root transformed (count data). The graph is divided into two panels, one for each *temperature* condition (ie. either "constant" or "fluctuating"). The colours within each panel refer to the two experiment types (see legend). The actual observations, the full dots, are rendered with some jittering and some transparency. In addition, regression lines are added to graph to connect the mean values of the different experiments type combinations.

## 8.7 All graphs together for scientific publication

First row rates, second row efficiencies.

```
ggarrange(gg.infection.rate_sp,
          gg.dissemination.rate_sp,
          gg.trasmission.rate_sp,
          ##
          gg.dissemination.efficiency_sp,
          gg.trasmission.efficiency_sp,
          common.legend = TRUE)
```

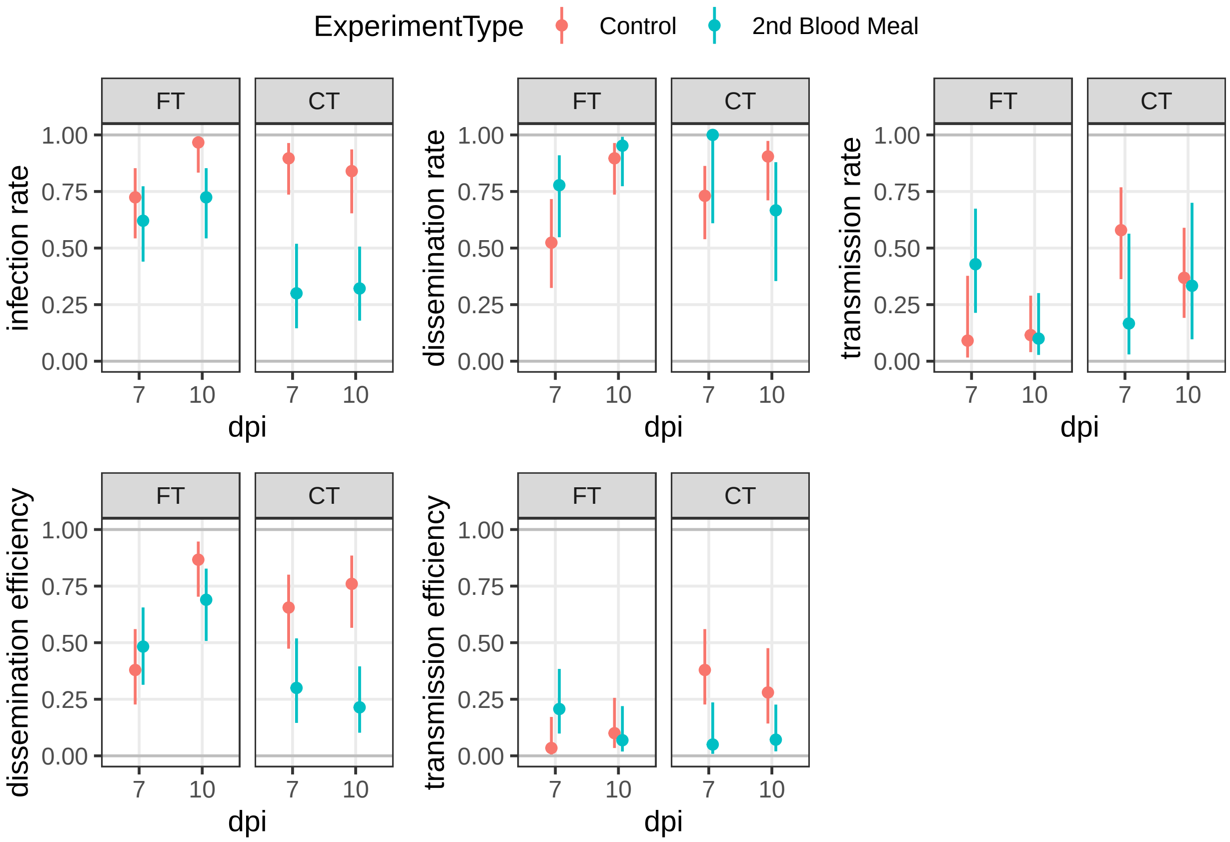

The upper row of graphs displays the results for the three rates (ie. infection, dissemination and transmission). The bottom row displays the results for the two efficiencies (dissemination and transmission). For each response variable a graph with two panels is created. The panel on the left refers to “constant” temperature conditions, while the panel on the right refers to “fluctuating” temperature conditions. Within each panel the colours refers to experiment type (see legend). On each graph the estimated probability is rendered as a full circle, while the its corresponding confidence interval is rendered as a vertical bar.

We also refer the reader to table 1 where all estimates and confidence intervals are reported.

## 8.8 Other graphs for the publication

We create a proto plot with no legend as wished.

```
gg.proto_sp_2 <- ggplot(
  data = d.vectorCompetence,
  mapping = aes(x = Time.fac,
                colour = ExperimentType)) +
  scale_y_continuous(limits = c(0,1), minor_breaks = NULL) +
  geom_hline(yintercept = c(0,1), colour = "gray") +
  geom_point(position = position_dodge(0.25),
             show.legend = FALSE) + ## !
  geom_linerange(position = position_dodge(0.25),
                 show.legend = FALSE) + ##  !
  facet_grid(.~Temperature.fac) +
  theme_bw() +
  xlab(label = "dpi")
```

### 8.8.1 Infection rate

```
gg.infection.rate_sp_FT <- gg.proto_sp_2 %+%
  filter(d.vectorCompetence, Temperature.fac == "FT") +
  aes(y = infection.rate,
      ymin = lower_infection.rate,
      ymax = upper_infection.rate) +
  ylab(label = "infection rate")
##
gg.infection.rate_sp_FT
```

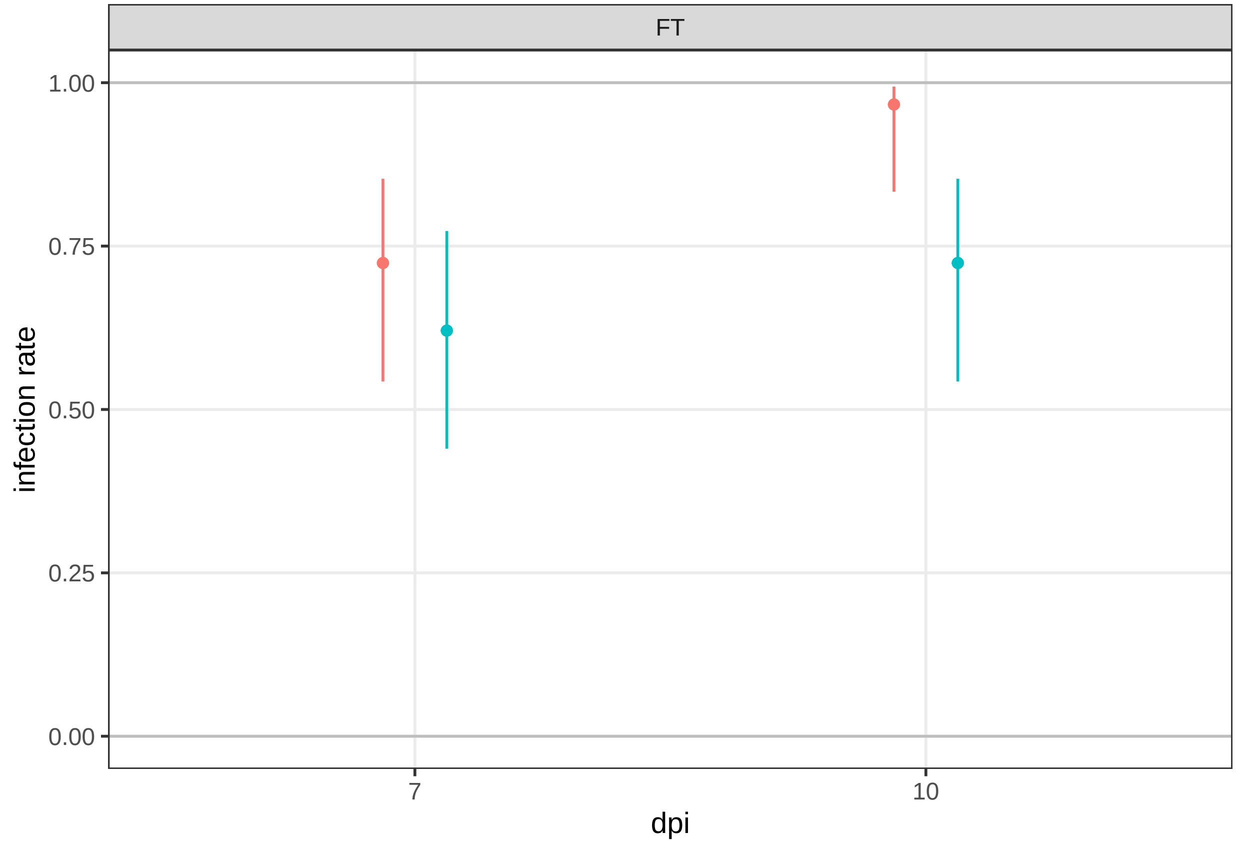


```
gg.infection.rate_sp_CT <- gg.proto_sp_2 %+%
  filter(d.vectorCompetence, Temperature.fac == "CT") +
  aes(y = infection.rate,
      ymin = lower_infection.rate,
      ymax = upper_infection.rate) +
  ylab(label = "infection rate")
##
gg.infection.rate_sp_CT
```

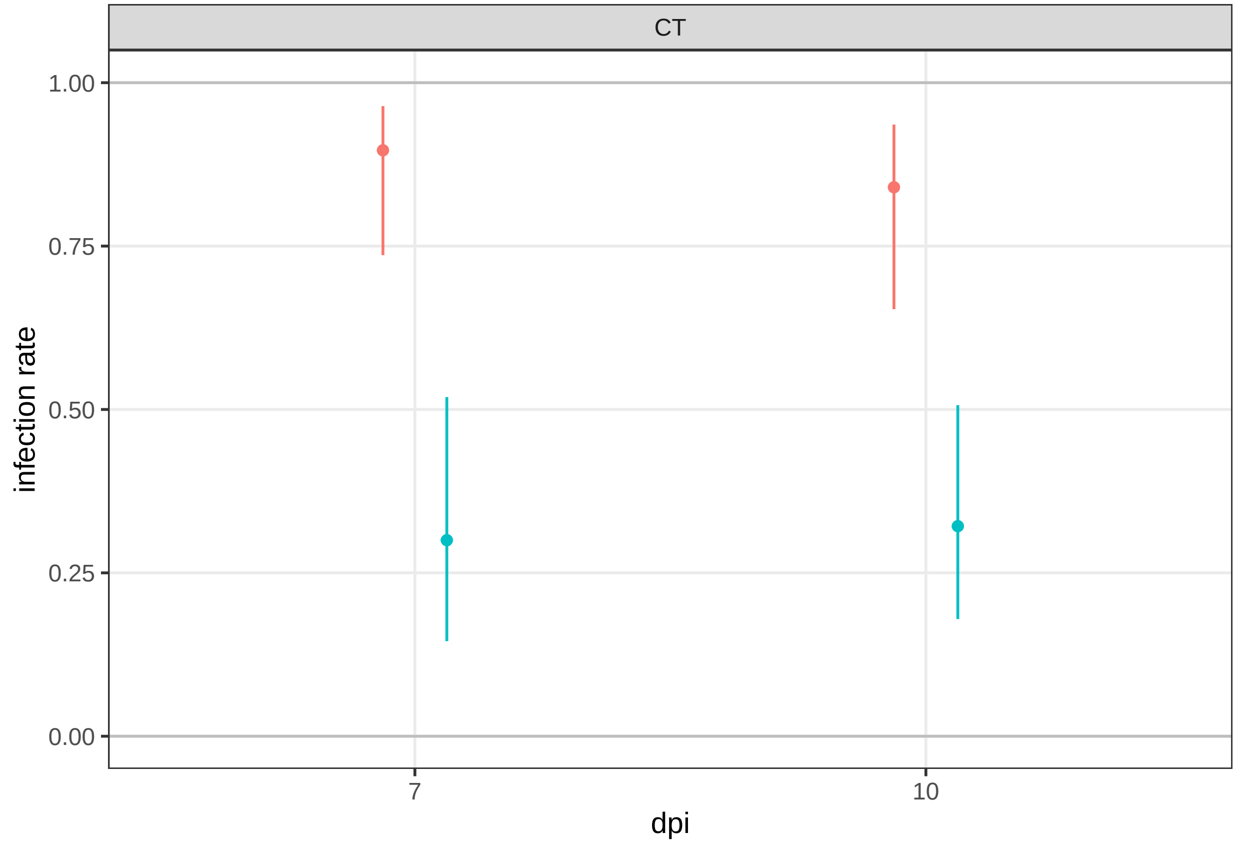


### 8.8.2 Dissemination efficiency

```
gg.dissemination.efficiency_sp_FT <- gg.proto_sp_2 %+%
    filter(d.vectorCompetence, Temperature.fac == "FT") +
  aes(y = dissemination.efficiency,
      ymin = lower_dissemination.efficiency,
      ymax = upper_dissemination.efficiency) +
  ylab(label = "dissemination efficiency")
##
gg.dissemination.efficiency_sp_FT
```

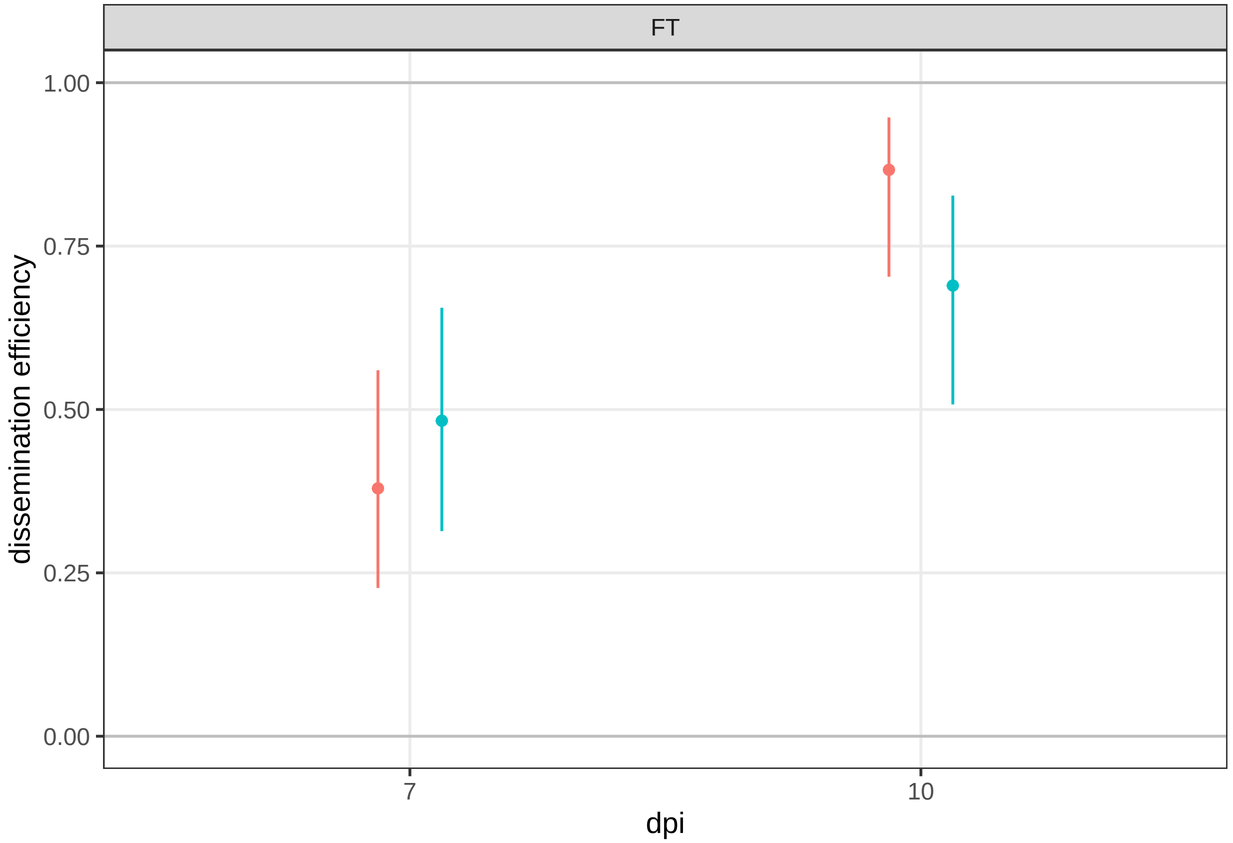


```
gg.dissemination.efficiency_sp_CT <- gg.proto_sp_2 %+%
    filter(d.vectorCompetence, Temperature.fac == "CT") +
  aes(y = dissemination.efficiency,
      ymin = lower_dissemination.efficiency,
      ymax = upper_dissemination.efficiency) +
  ylab(label = "dissemination efficiency")
##
gg.dissemination.efficiency_sp_CT
```

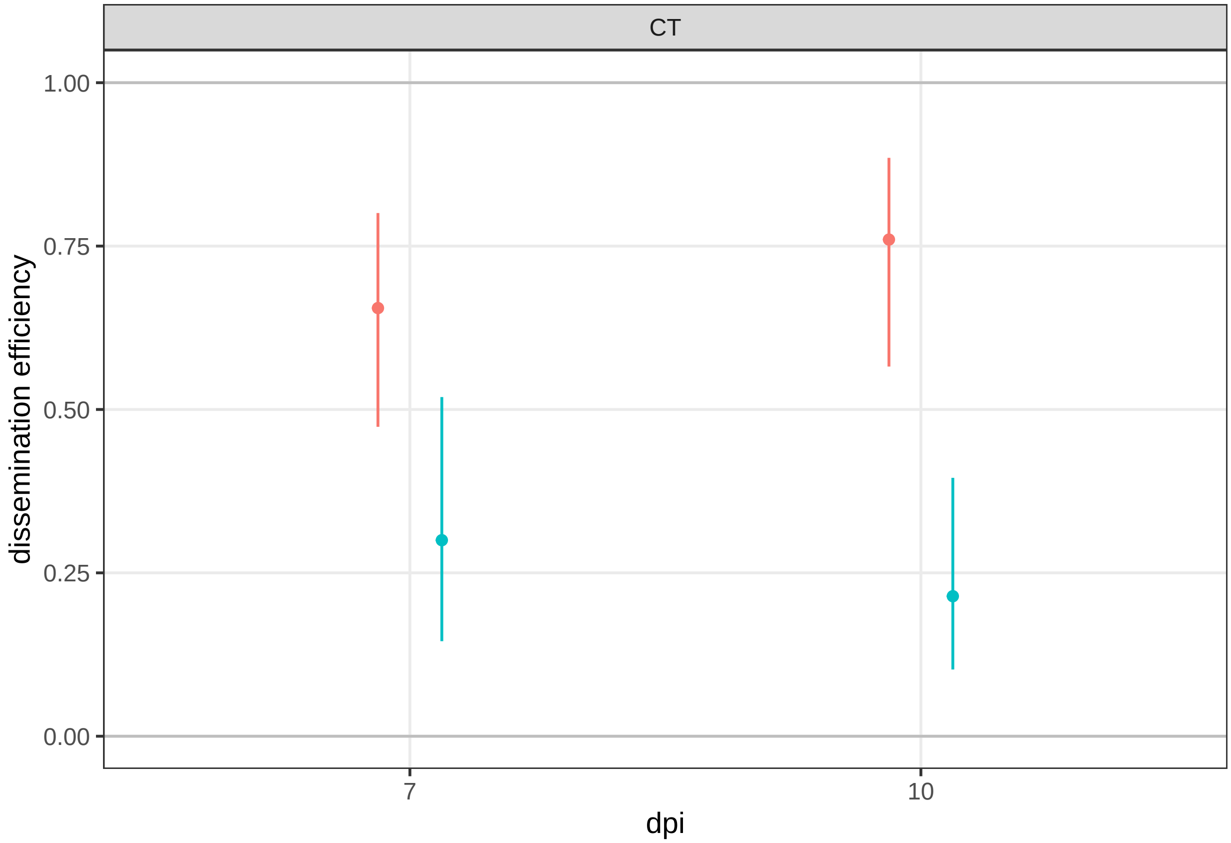


#### 8.8.3 Dissemination rate

```
gg.dissemination.rate_sp_FT <- gg.proto_sp_2  %+%
    filter(d.vectorCompetence, Temperature.fac == "FT") +
  aes(y = dissemination.rate,
      ymin = lower_dissemination.rate,
      ymax = upper_dissemination.rate) +
  ylab(label = "dissemination rate")
##
gg.dissemination.rate_sp_FT
```

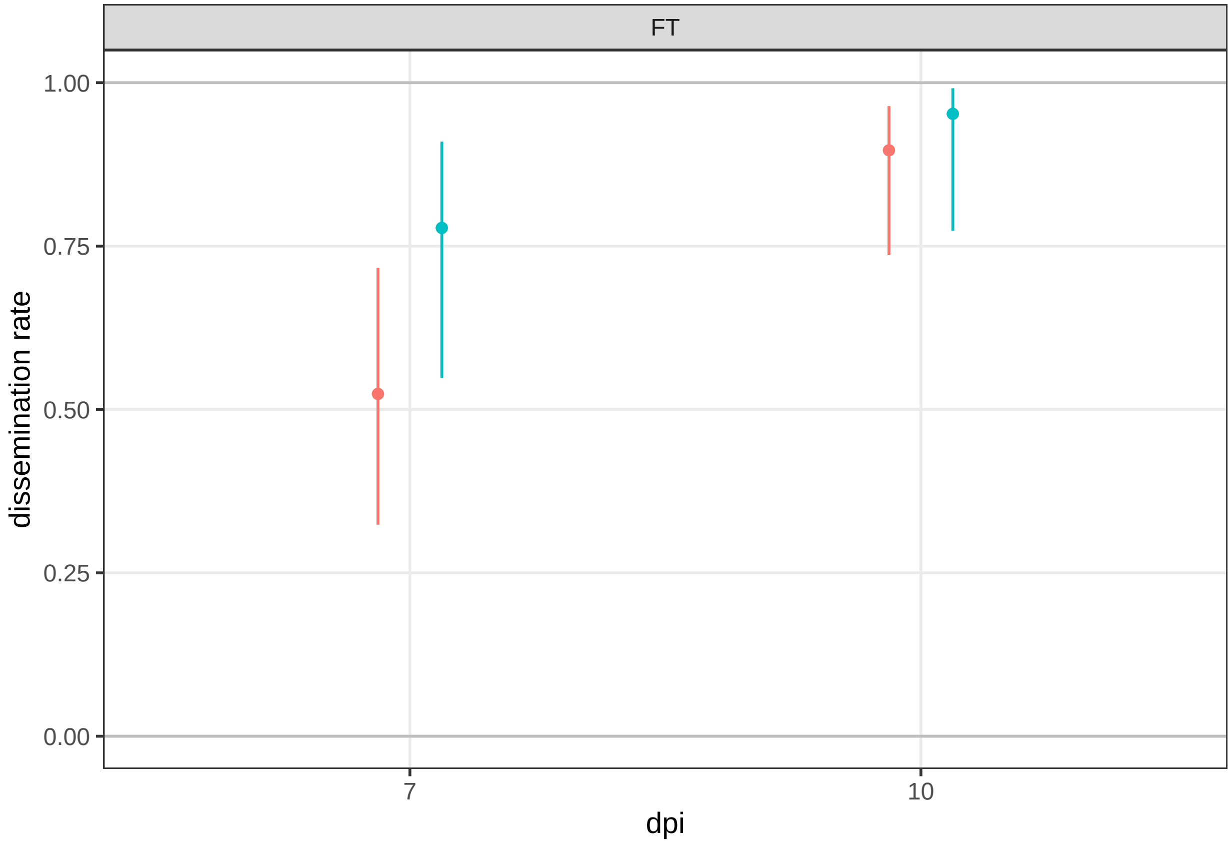


```
gg.dissemination.rate_sp_CT <- gg.proto_sp_2  %+%
    filter(d.vectorCompetence, Temperature.fac == "CT") +
  aes(y = dissemination.rate,
      ymin = lower_dissemination.rate,
      ymax = upper_dissemination.rate) +
  ylab(label = "dissemination rate")
##
gg.dissemination.rate_sp_CT
```

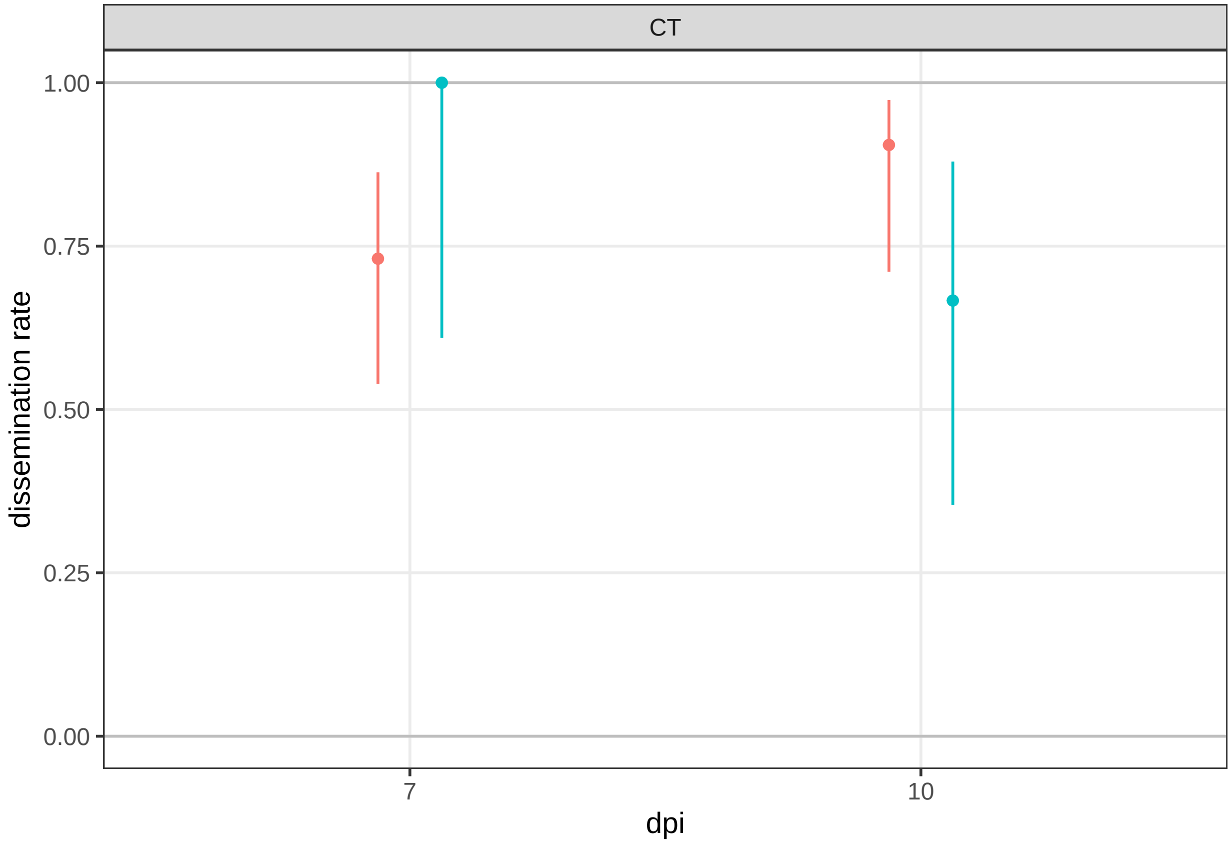


#### 8.8.4 Transmission efficiency

```
gg.trasmission.efficiency_sp_FT <- gg.proto_sp_2 %+%
    filter(d.vectorCompetence, Temperature.fac == "FT") +
  aes(y = transmission.efficiency,
      ymin = lower_transmission.efficiency,
      ymax = upper_transmission.efficiency) +
  ylab(label = "transmission efficiency")
##
gg.trasmission.efficiency_sp_FT
```

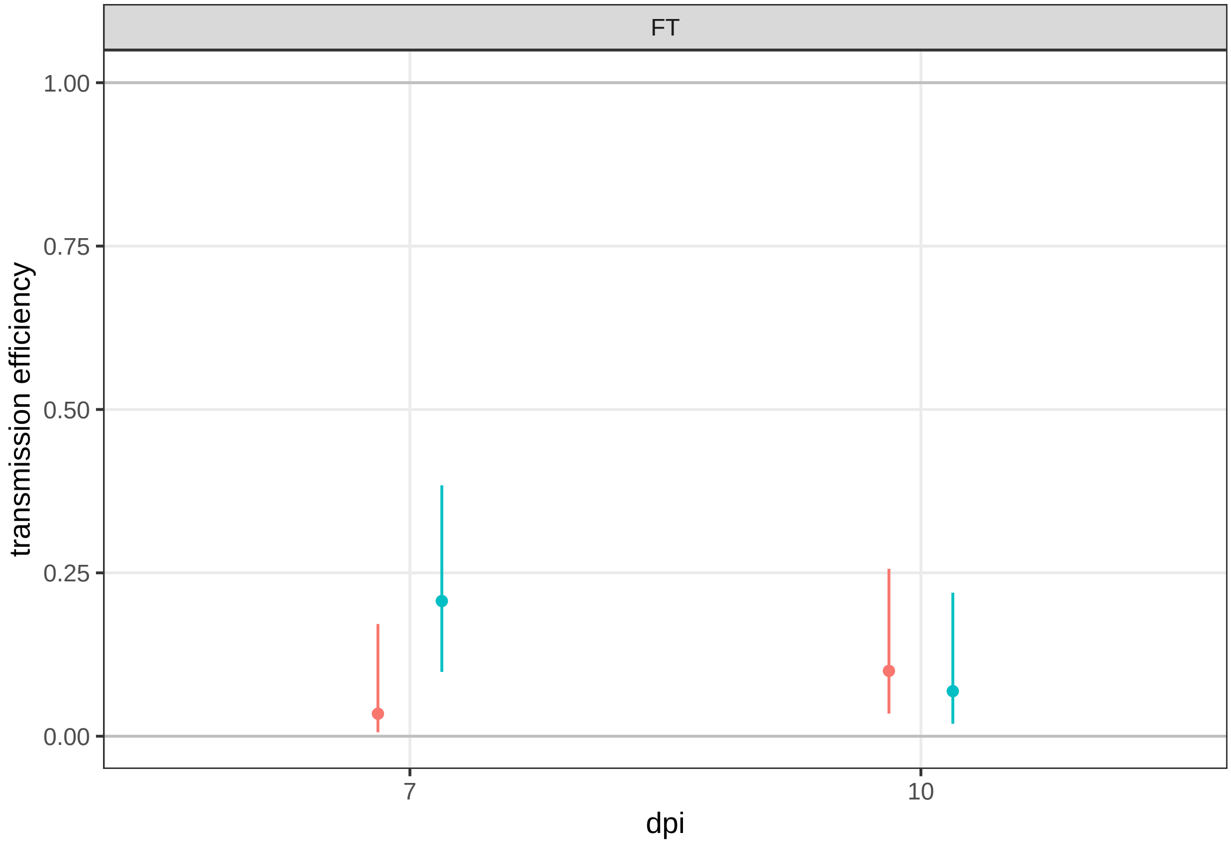


```
gg.trasmission.efficiency_sp_CT <- gg.proto_sp_2 %+%
    filter(d.vectorCompetence, Temperature.fac == "CT") +
  aes(y = transmission.efficiency,
      ymin = lower_transmission.efficiency,
      ymax = upper_transmission.efficiency) +
  ylab(label = "transmission efficiency")
##
gg.trasmission.efficiency_sp_CT
```

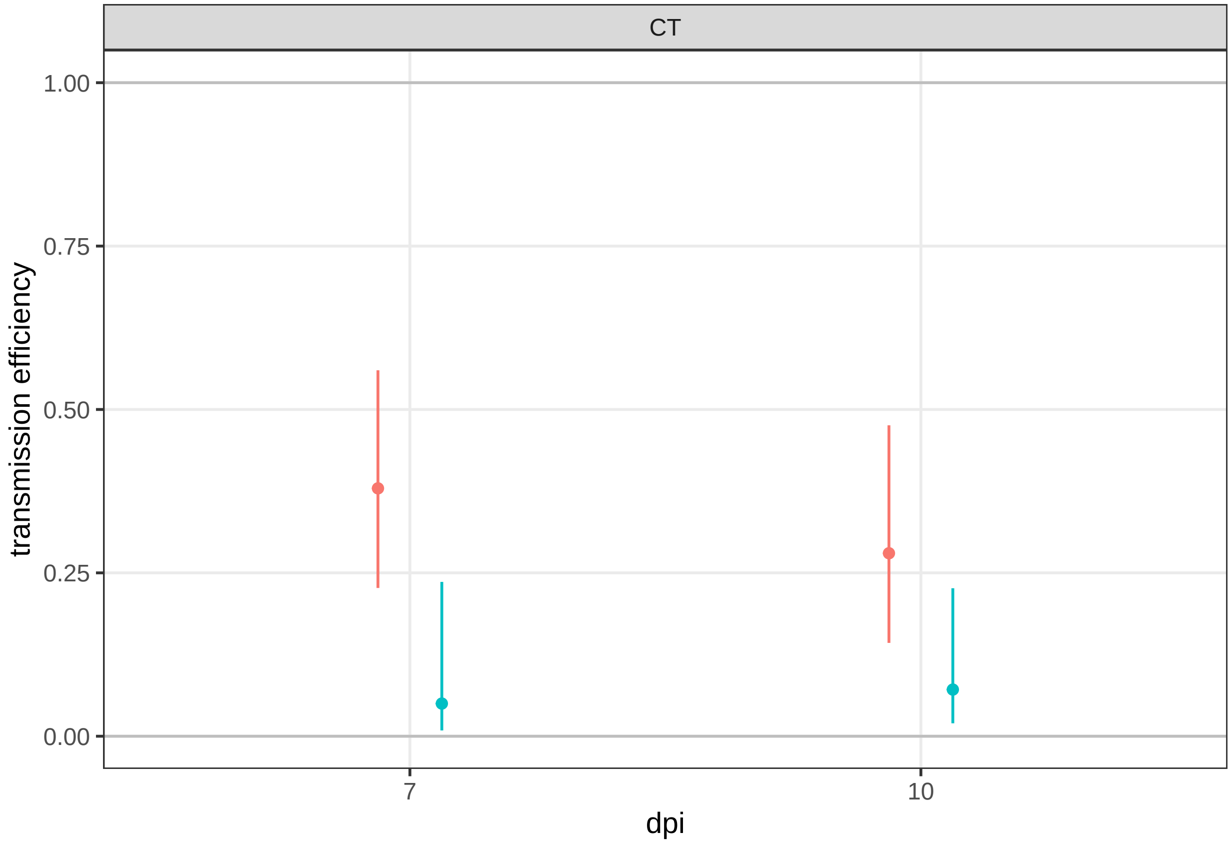


#### 8.8.5 Transmission rate

```
gg.trasmission.rate_sp_FT <- gg.proto_sp_2 %+%
    filter(d.vectorCompetence, Temperature.fac == "FT") +
  aes(y = transmission.rate,
      ymin = lower_transmission.rate,
      ymax = upper_transmission.rate) +
  ylab(label = "transmission rate")
##
gg.trasmission.rate_sp_FT
```

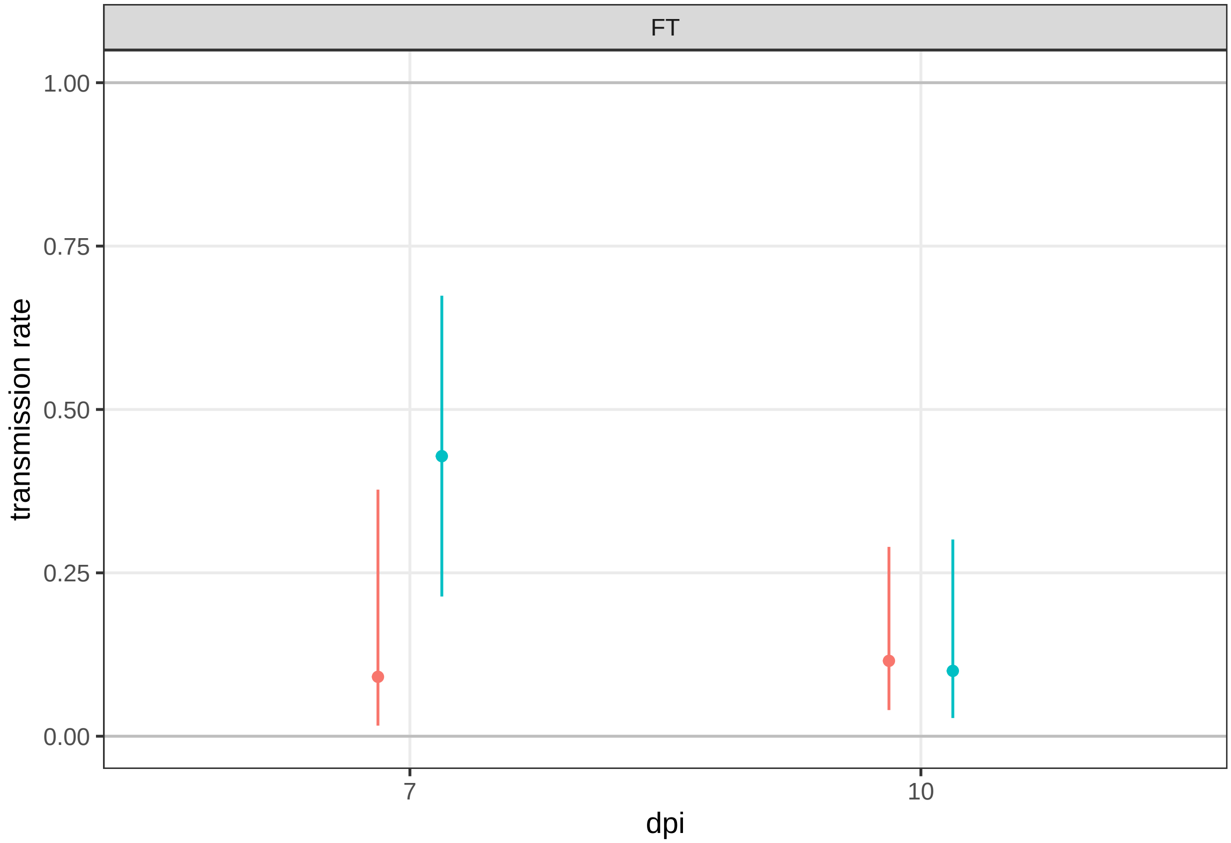


```
gg.trasmission.rate_sp_CT <- gg.proto_sp_2 %+%
    filter(d.vectorCompetence, Temperature.fac == "CT") +
  aes(y = transmission.rate,
      ymin = lower_transmission.rate,
      ymax = upper_transmission.rate) +
  ylab(label = "transmission rate")
##
gg.trasmission.rate_sp_CT
```

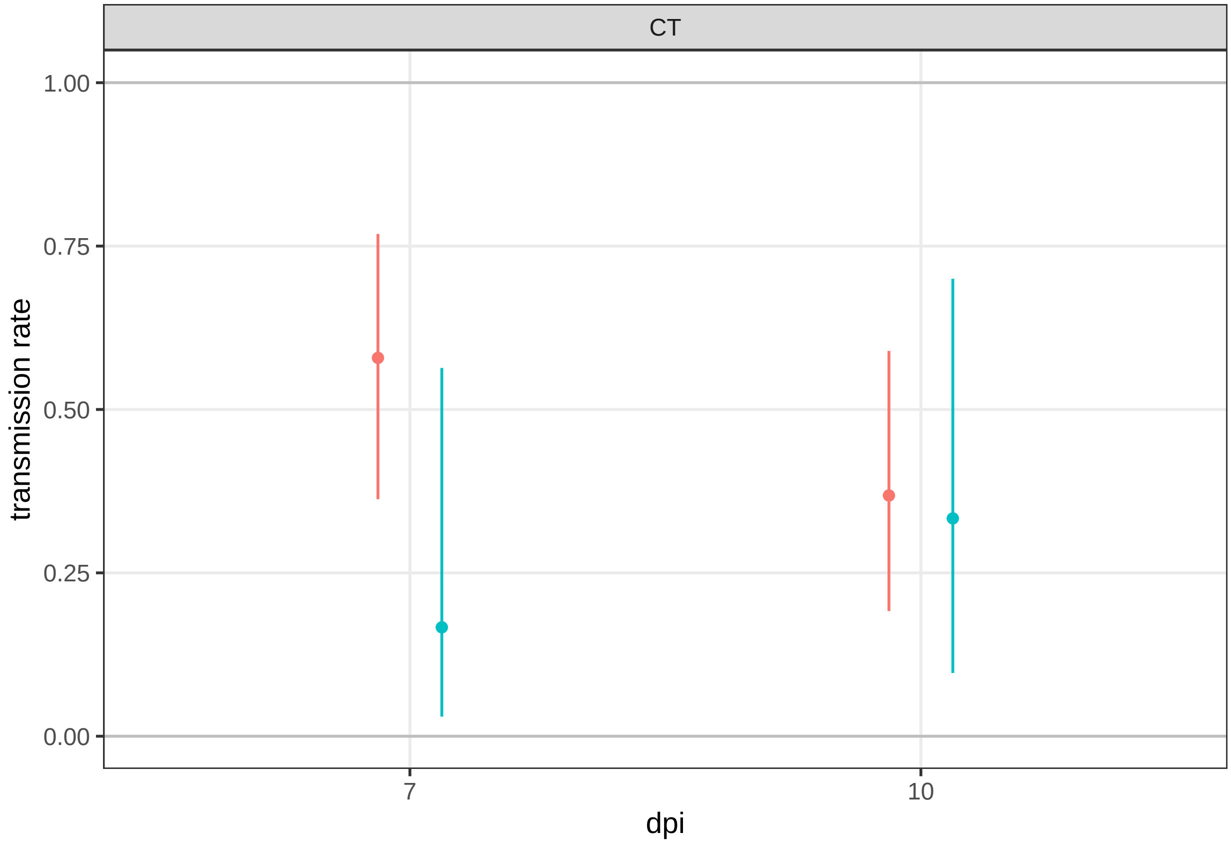


## 8.9 Plaque forming units

To be able to arrange the graphs as done “manually” we need to relevel the *Temperature.fac* factor.

```
d.PFU <- d.PFU %>%
  mutate(Temperature.fac_rel = relevel(Temperature.fac,
                                       ref = "CT" ))
```

```
gg.PFU_sp_2 <- ggplot(data = d.PFU,
       mapping = aes(y = PFU.num,
                     x = Time,
                     colour = ExperimentType)) +
  scale_y_sqrt() +
  geom_hline(yintercept = 0, col = "gray") +
  geom_jitter(alpha = 0.2, width = 0.1, height = 0.1,
              show.legend = FALSE) +
  facet_grid(~Temperature.fac_rel) +
  stat_summary(fun = mean,
               geom = "line",
               show.legend = FALSE) +
  theme_bw()
##
gg.PFU_sp_2
```

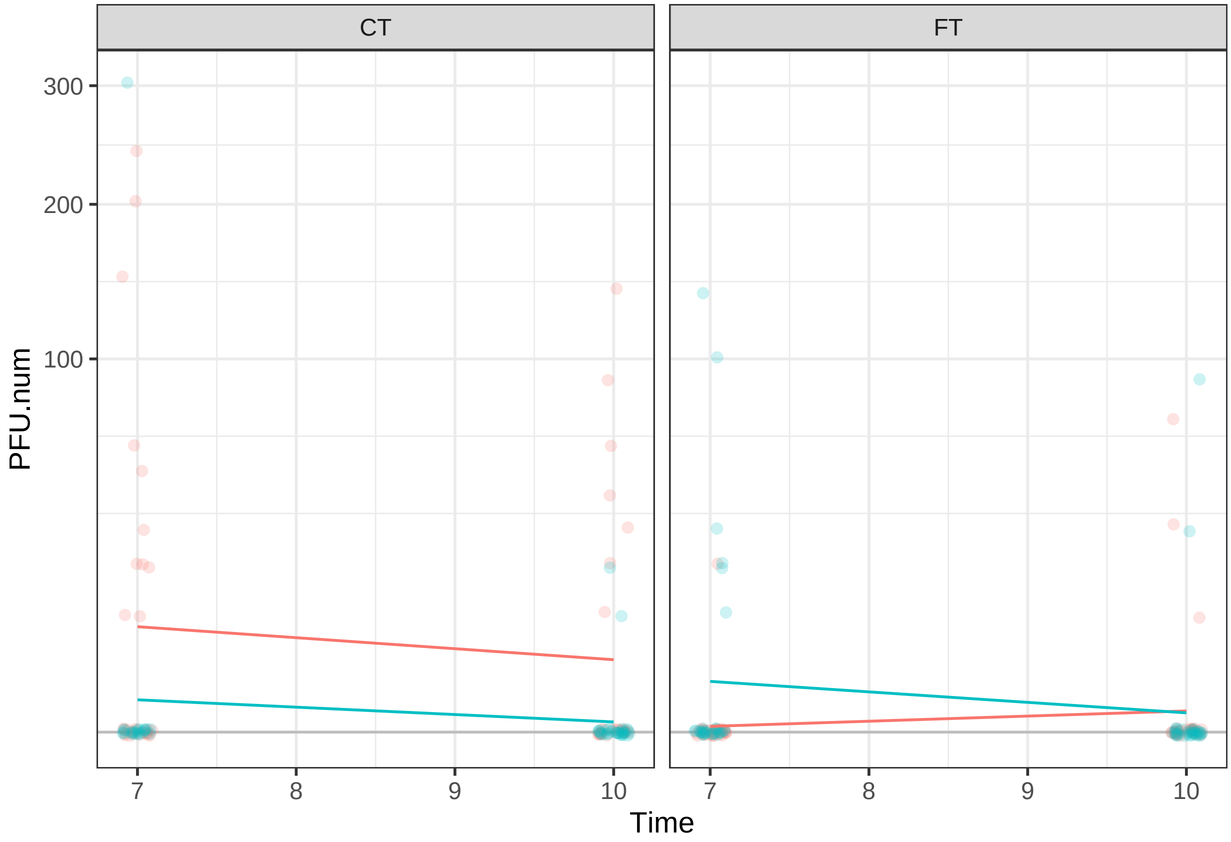


## 8.10 Putting the graphs together

We start with all FT.

```
ggarrange(gg.infection.rate_sp_FT,
          gg.dissemination.rate_sp_FT,
          gg.trasmission.rate_sp_FT,
          ##
          gg.dissemination.efficiency_sp_FT,
          gg.trasmission.efficiency_sp_FT)
```

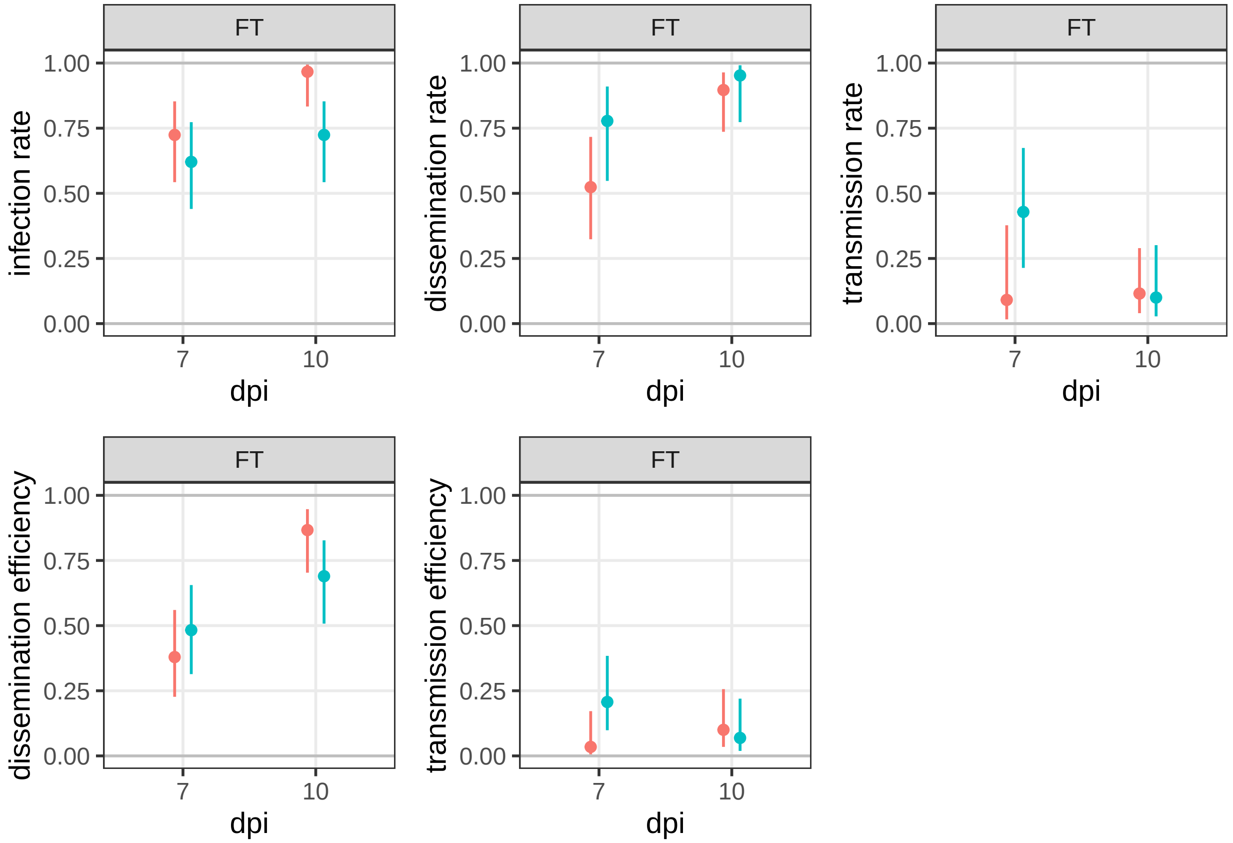


We do the same for CT.

```
ggarrange(gg.infection.rate_sp_CT,
          gg.dissemination.rate_sp_CT,
          gg.trasmission.rate_sp_CT,
          ##
          gg.dissemination.efficiency_sp_CT,
          gg.trasmission.efficiency_sp_CT)
```

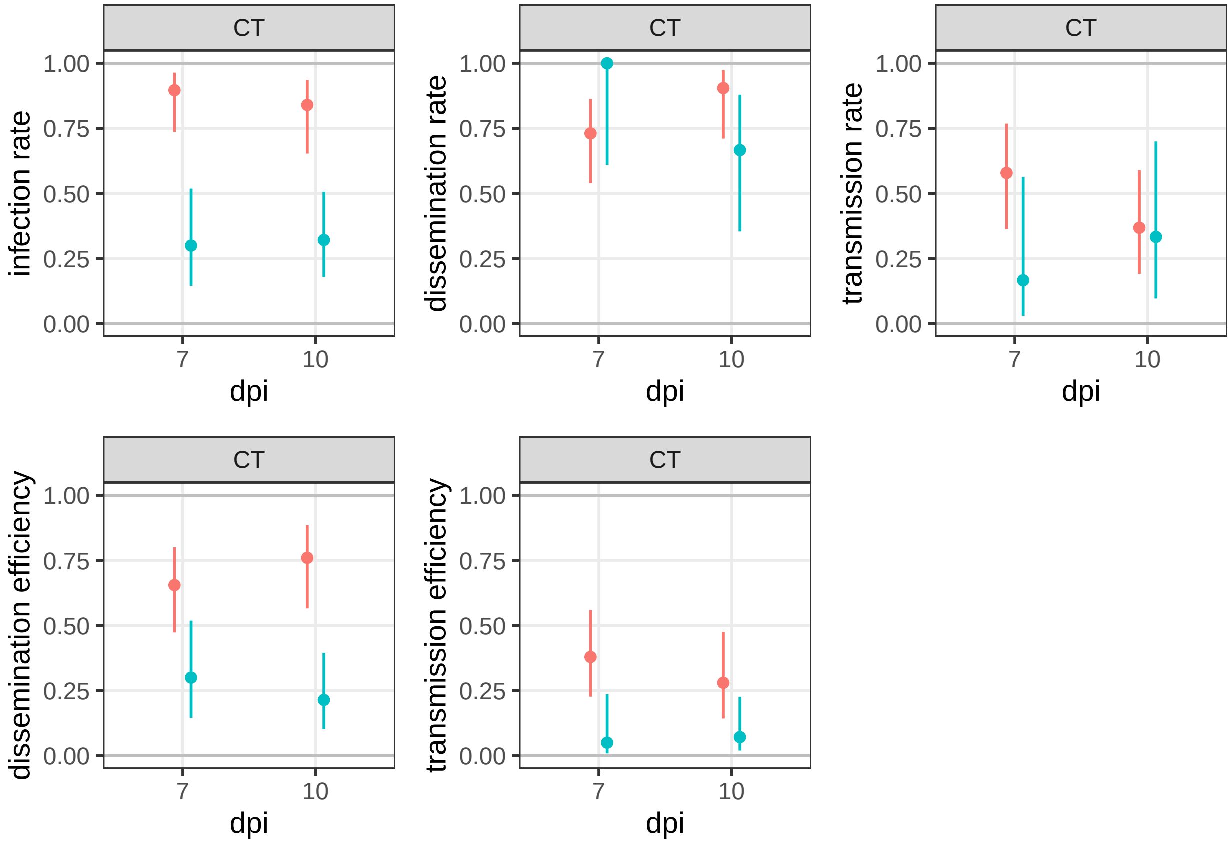
CT
infection rate
dissemination rate
transmission rate
dissemination efficiency
transmission efficiency
1.00
0.75
0.50
0.25
0.00
7
10
dpi

# 9 Session Information

```
sessionInfo()
```

```
R version 4.2.3 (2023-03-15 ucrt)
Platform: x86_64-w64-mingw32/x64 (64-bit)
Running under: Windows 10 x64 (build 22621)

Matrix products: default

locale:
[1] LC_COLLATE=English_Switzerland.utf8  LC_CTYPE=English_Switzerland.utf8
[3] LC_MONETARY=English_Switzerland.utf8 LC_NUMERIC=C
[5] LC_TIME=English_Switzerland.utf8

attached base packages:
[1] stats     graphics  grDevices utils     datasets  methods   base

other attached packages:
[1] ggpubr_0.4.0     binom_1.1-1.1    ggplot2_3.3.6    dplyr_1.0.9
[5] checkpoint_1.0.2 knitr_1.39

loaded via a namespace (and not attached):
 [1] pillar_1.7.0      compiler_4.2.3    tools_4.2.3       digest_0.6.29
 [5] evaluate_0.15     lifecycle_1.0.1   tibble_3.1.6      gtable_0.3.0
 [9] pkgconfig_2.0.3   rlang_1.0.2       cli_3.3.0         rstudioapi_0.13
[13] yaml_2.3.5        xfun_0.30         fastmap_1.1.0     gridExtra_2.3
[17] withr_2.5.0       stringr_1.4.0     generics_0.1.2    vctrs_0.4.1
[21] cowplot_1.1.1     grid_4.2.3        tidyselect_1.1.2 glue_1.6.2
[25] R6_2.5.1          rstatix_0.7.0     fansi_1.0.3       rmarkdown_2.14
[29] carData_3.0-5     farver_2.1.0      car_3.0-12        tidyr_1.2.0
[33] purrr_0.3.4       magrittr_2.0.3    backports_1.4.1   scales_1.2.0
[37] ellipsis_0.3.2    htmltools_0.5.2   abind_1.4-5       colorspace_2.0-3
[41] ggsignif_0.6.3    labeling_0.4.2    utf8_1.2.2        stringi_1.7.6
[45] munsell_0.5.0     broom_0.8.0       crayon_1.5.1
```
